# CNS function and dysfunction during exposure to hyperbaric oxygen in operational and clinical settings

**DOI:** 10.1016/j.redox.2019.101159

**Published:** 2019-03-09

**Authors:** Geoffrey E. Ciarlone, Christopher M. Hinojo, Nicole M. Stavitzski, Jay B. Dean

**Affiliations:** a Undersea Medicine Department, Naval Medical Research Center, 503 Robert Grant Ave., Silver Spring, MD, USA; b Department of Molecular Pharmacology and Physiology, Hyperbaric Biomedical Research Laboratory, Morsani College of Medicine, University of South Florida, Tampa, FL, USA

**Keywords:** CNS oxygen toxicity, Hyperoxia, Reactive oxygen and nitrogen species, Brainstem, Seizure genesis, Seizure mitigation, AED, anti-epileptic drugs, ATA, atmospheres absolute pressure, CBF, cerebral blood flow, CN, cranial nerve, CNS, central nervous system, CNS-OT, central nervous system oxygen toxicity, CO_2_, carbon dioxide, cSC, caudal Solitary Complex, DCS, decompression sickness, DHE, dihydroethidium, DISSUB, disabled submarine, E2, estradiol, F_I_O_2_, fractional concentration of inspired oxygen, fsw, feet of sea water, GABA, Gamma-Aminobutyric Acid, Glu, glutamate, HBO_2_, hyperbaric oxygen, HBO_2_-PC, hyperbaric oxygen preconditioning, HBOT, hyperbaric oxygen therapy, LOC, loss of consciousness, mm Hg, millimeters of Mercury, N_2_, nitrogen, NADPH, Nicotinamide adenine dinucleotide phosphate, NMDA, N-methyl-d-aspartate, NTS, nucleus tractus solitarius, O_2_, oxygen, OxIP, oxygen-induced potentiation, P_B_, barometric pressure, PCO_2_, partial pressure of carbon dioxide, PO_2_, partial pressure of oxygen, P_I_O_2_, partial pressure of inspired oxygen, RN, Royal Navy, RONS, reactive oxygen and nitrogen species, S/Sx, signs and symptoms, USN, United States Navy, VENTID-C, Vison-Ears-Nausea-Twitching/Tingling-Irritability-Dizziness-Convulsions

## Abstract

Hyperbaric oxygen (HBO_2_) is breathed during hyperbaric oxygen therapy and during certain undersea pursuits in diving and submarine operations. What limits exposure to HBO_2_ in these situations is the acute onset of central nervous system oxygen toxicity (CNS-OT) following a latent period of safe oxygen breathing. CNS-OT presents as various non-convulsive signs and symptoms, many of which appear to be of brainstem origin involving cranial nerve nuclei and autonomic and cardiorespiratory centers, which ultimately spread to higher cortical centers and terminate as generalized tonic-clonic seizures. The initial safe latent period makes the use of HBO_2_ practical in hyperbaric and undersea medicine; however, the latent period is highly variable between individuals and within the same individual on different days, making it difficult to predict onset of toxic indications. Consequently, currently accepted guidelines for safe HBO_2_ exposure are highly conservative. This review examines the disorder of CNS-OT and summarizes current ideas on its underlying pathophysiology, including specific areas of the CNS and fundamental neural and redox signaling mechanisms that are thought to be involved in seizure genesis and propagation. In addition, conditions that accelerate the onset of seizures are discussed, as are current mitigation strategies under investigation for neuroprotection against redox stress while breathing HBO_2_ that extend the latent period, thus enabling safer and longer exposures for diving and medical therapies.

“*To Paul Bert (1833–1886), the great French physiologist, is due the foundation of our knowledge of respiration at high and low pressures, and at low and high tensions of oxygen. His research and practical experimental work proved the dangers of breathing pure oxygen above a certain pressure … But only since the development of deep diving and aviation have his genius and work been fully recognized and appreciated … All later physiologists who have worked in this field of research, designers of breathing and diving appliances and men who fly at great altitudes or go deep under the sea, are his debtors*” [65].

## 1 Atmospheric and brain PO_2_: defining normoxia and hypoxia versus hyperoxia

We live at the bottom of a sea of gas that is comprised mainly of 21% oxygen (O_2_) and 78% nitrogen (N_2_). For reference, the partial pressure of O_2_ (PO_2_) at sea level is 0.21 atm absolute (ATA), which is defined by the fractional concentration of inspired O_2_ in air (F_I_O_2_ = 0.21) multiplied by the barometric pressure (P_B_; i.e., 1 ATA).^1^ This is normobaric air or simply, normoxia. Atmospheric PO_2_ has remained relatively constant since the origin of animals and plants through a net balance between O_2_ production via oxygenic photosynthesis and photochemical dissociation of water, and O_2_ removal via animal respiration, decay of biological materials, formation of water, and oxidation of Earth's surface minerals [9,29,164]. Normoxia can also be defined in terms of the PO_2_ levels that are measured in various body compartments while inspiring normobaric air; for example, terrestrial mammals inspiring 0.21 ATA O_2_ have an average alveolar PO_2_ of ∼100 mm of Mercury (mm Hg), a systemic arterial PO_2_ of ∼90 mm Hg, a neural extracellular PO_2_ ranging from <10 to 35 mm Hg, and an intracellular/mitochondrial PO_2_ varying from ∼1 to 3 mm Hg [68,139]. This is the level of oxygenation under which the mammalian central nervous system (CNS) has evolved and currently functions.

The mammalian CNS is also highly sensitive to hypoxia, a decrease in inspired PO_2_ (P_I_O_2_) that occurs during ascent to higher terrestrial altitudes (hypobaric hypoxia) or during a reduction in alveolar PO_2_ during hypoventilation near sea level. Likewise, reoxygenation following a hypoxic episode creates a transient relatively hyperoxic event that has powerful, long lasting effects on neural function [96,154]. Exposure to hypobaric hypoxia or alveolar hypoventilation endangers adequate oxygenation of blood passing through the pulmonary circulation. To maintain oxygenation during hypoxia, mammals have evolved a powerful brainstem-controlled integrated cardiorespiratory reflex to ensure adequate arterial and tissue oxygenation [102,154]. Thus, normoxia and modest levels of hypoxia followed by reoxygenation are commonly occurring conditions of life to which the CNS adapts and functions.

By contrast, hyperoxia, which is defined as P_I_O_2_ >0.21 ATA, is an unnatural condition that only occurs through human interventions that enable inhalation of an artificial atmosphere of O_2_-enriched gas mixture at normobaric (room) pressure and hyperbaric pressure (P_B_ > 1 ATA, where P_I_O_2_ > 1 ATA). Thus, during exposure to a hyperoxic gas mixture, oxidative signaling mechanisms in the mammalian CNS [90] are abnormally activated. Moreover, there is no physiological hyperoxic ventilatory response that compensates for arterial hyperoxemia. In fact, protracted breathing of an extreme hyperoxic gas mixture at P_I_O_2_ >1 ATA O_2_ (i.e., hyperbaric oxygen, HBO_2_) activates a variety of anomalous cardiorespiratory responses that include transient bradycardia followed by tachycardia, hypertension, paradoxical hyperventilation, and other respiratory abnormalities such as coughing, dyspnea, and spasms of the diaphragm and upper airway that precede onset of tonic-clonic seizures [69,87,152]. Collectively, these non-convulsive signs and symptoms (S/Sx) that terminate in tonic-clonic seizures comprise the malady known as CNS oxygen toxicity (CNS-OT). Remarkably, despite the risk of CNS-OT, breathing HBO_2_ has several benefits in the context of hyperbaric, diving, and submarine medicine due to the safe latent period that precedes onset of any debilitating “acute toxic end-points” [87,191]. Thus, breathing HBO_2_, which is an unnatural stimulus for the CNS, has become a commonly encountered condition of life.

Accordingly, the six goals of this review article are as follows: 1) to summarize the conditions under which humans breathe a hyperoxic atmosphere, focusing on the use of HBO_2_ in undersea physiology and medicine and hyperbaric oxygen therapy (HBOT); 2) summarize the pathophysiology and wide-ranging S/Sx that define CNS-OT, and review conditions that accelerate onset of CNS-OT; 3) discuss the regions of the brain that are thought to be involved in seizure genesis and propagation; 4) summarize current ideas on the underlying neural mechanisms that cause CNS-OT; 5) review current mitigation strategies under study that delay onset of CNS-OT to enable longer, deeper, and safer dives; and 6) outline recommendations for future research on neuroprotection against CNS-OT.

## 2 Why do humans breathe O_2_-enriched, abnormal atmospheres?

Hyperbaric hyperoxia, despite being an unnatural and manmade phenomenon, is a frequently encountered stimulus in HBOT for treating conditions such as late radiation tissue injury and problematic wounds, as well as resolving disorders caused by inert gas bubbles and emboli [181]. Hyperbaric hyperoxia also enables specialized undersea activities covered under recreational, commercial, and military diving operations [32,86], and pressurized disabled submarine (DISSUB) emergencies that require escape or rescue [101,133,182]. In these contexts, the greatest benefit of using O_2_-enriched gas mixtures or pure O_2_ when diving is the avoidance of maladies that involve inert gases. For example, when gases such as N_2_ or helium are inhaled at increased pressures for prolonged periods, they dissolve into peripheral tissues in accordance with Henry's Law and begin to incur a decompression obligation, meaning that on ascent the individual must allow adequate time for said gases to diffuse from the body's tissues and return to the lungs for exhalation. If ascent to the surface (decompression) occurs too quickly, supersaturation and bubble formation occur in vascular and extravascular tissues and result in Decompression Sickness (DCS), symptoms of which include joint pain, as well as more serious neurological and cardiopulmonary indications [144]. Breathing a hyperoxic gas mixture prior to decompression, a strategy known as oxygen prebreathing or isobaric denitrogenation, can be used to decrease or omit decompression obligations and avert the risk for DCS not only in divers completing prolonged dive profiles, but also in pressurized DISSUB personnel who are exposed to hyperbaric air inside a compromised submarine at a maximum rescuable depth equivalent of 5 ATA ambient pressure [101,133,182]. Finally, high dose O_2_ can be used in conjunction with closed-circuit underwater breathing apparatus (e.g., MK-25 and LAR V Dräger rebreathers) in military clandestine operations to maintain stealth (no bubbles are released during exhalation) during shallow transit [86,144,191].

Clinically, HBO_2_ is highly efficacious in the treatment of several conditions spanning a broad pathological range [145]. In clinical and hospital settings, HBOT requires the use of a single- or multi-place hyperbaric chamber to elevate P_B_. Patients in a multi-place chamber wear an HBOT hood or mask that is ventilated with 100% O_2_ while the primary chamber is pressurized in parallel with air. To have a therapeutic effect, HBOT requires the patient inhale 100% O_2_ at a pressure of no less than 1.4 ATA. HBOT treatments will generally involve pressurization up to 3 ATA, interspersed by periodic air breaks. For example, several 20–30 min exposures to HBO_2_ are interspersed with 5–10 min air breaks to avert toxic S/Sx of CNS and pulmonary O_2_ toxicity [42,181]. These depth and time limitations are set for patient safety during any given session of HBOT. Many of the chronic conditions treated need repeated HBO_2_ sessions over several weeks in combination with other measures to stimulate wound healing. In the United States there are currently 14 indications that are approved for treatment with HBO_2_ [181]. Two such indications are DCS and arterial gas embolism, collectively referred to as decompression illness, which can occur in divers who do not allot adequate time during decompression for the elimination of excess inert gas and results in the formation of bubbles that cause pain and occlusion of vasculature, the latter resulting in tissue hypoxia. In these cases, treatment with pressure and O_2_-enriched gas mixture serve to decrease bubble size, thus reducing pain and restoring blood flow, while also correcting tissue hypoxia and inducing a large inert gas gradient to expedite washout. Several indications, such as anemia, carbon monoxide poisoning, and tissue injuries also require hypoxia correction and others, such as reperfusion complications following crush or ischemia, benefit from HBOT due to inhibition of neutrophil adhesion and subsequent vasoconstriction [145,181].

## 3 The toxic effects of oxygen

### 3.1 Poisoning by O_2_: a historical perspective of CNS-OT

The use of HBO_2_ in the foregoing situations is limited by the risk of CNS-OT, which was discovered by Paul Bert (1833–1886; pronounced “Bear”) who reported that “… compressed air acts only by the tension of the oxygen which it contains, and that this oxygen can kill animals rapidly with convulsive symptoms … resulting from what I shall call, if only for convenience in nomenclature, poisoning by oxygen” [18]. Bert, a student of the great physiologist, Claude Bernard (1813–1878), determined that “oxygen poisoning” occurred in invertebrate and vertebrate animals, including both non-mammalian and mammalian species. In recognition of Bert's discovery, O_2_ poisoning of the brain to the point of losing consciousness and developing tonic-clonic seizures is also called the “Paul Bert Effect” [9]. Paul Bert is widely acknowledged as the scientific founder of barophysiology and credited with discovering the basic physiology underlying many of the maladies that arise from protracted exposure to abnormal levels of P_B_ and inspired gases comprised of O_2_ (hypobaric hypoxia, CNS-OT), N_2_ (diving DCS), and carbon dioxide (CO_2_ narcosis/toxicity). Bert's original discoveries, which were published in *La pression barométrique—Recherches de physiologie expérimentelle* [18], are recognized as the foundation for today's environmental physiological specialties of aerospace, diving, and submarine physiology and medicine [65], and HBOT [58].^2^

The first human encounters with prolonged exposure to HBO_2_ occurred during 1910–1941. These early O_2_ dives were simulated in dry hyperbaric chambers while the volunteers sat quietly breathing either compressed air (F_I_O_2_ = 0.21) [1,89] or pure O_2_ (F_I_O_2_ ≅1.0) at raised P_B_ [16,17]. During this initial era of “oxtox” research, only twelve human exposures were documented in which acute toxic symptoms of O_2_ poisoning occurred [89]. Oxygen toxicity, however, became an important problem during World War II when Italian, British, and American combat divers began breathing pure O_2_ (F_I_O_2_ ≅1.0) using early versions of closed-circuit underwater rebreathers during covert diving operations [1,87,184]. At that time, Royal Navy (RN) and United States Navy (USN) undersea physiologists, based on the limited evidence, believed that humans could safely breathe pure O_2_ at 33  feet of sea water (fsw; 2 ATA) for 3 h, 66 fsw (3 ATA) for 2 h, and 99 fsw (4 ATA) for 30 min [16,17]. What was not known at that time, however, was that immersion in water increases the risk for CNS-OT seizures, cutting the latency time to seizures and loss of consciousness (LOC) by more than half compared to simulated dives in dry, gas-filled hyperbaric chambers [[87], [88], [89],^191^]; see below, *Risk factors for CNS-OT*. Consequently, by April 1942, numerous cases of HBO_2_ “hits” were documented in Britain's RN combat divers while O_2_-breathing with rebreathers at depths and durations that were previously thought to be safe [88]. To resolve the uncertainty of safe O_2_ limits for diving operations, the RN [88,89] and USN [191] initiated studies on the limits for human tolerance for HBO_2_ as a function of depth and exposure conditions, including HBO_2_-breathing under dry versus wet compression, and while resting or performing exercise. In England, these groundbreaking dive trials were led by Dr. Kenneth W. Donald (1911–1994), medical director of the RN's Admiralty Experimental Diving Unit, who oversaw a series of over 2000 man dives using RN volunteers [1], many of who subsequently received gallantry awards for their heroic exploits. Fifty years later, Dr. Donald summarized the findings of the RN and the USN in “Oxygen and the Diver” [86], stating that because of the medical dangers of suffering LOC and seizures while submerged, “It is very doubtful whether experimental diving on oxygen of large groups to ‘acute toxic end-points’ will ever be undertaken again …”.

### 3.2 Signs and symptoms of CNS-OT

#### 3.2.1 Seizures with loss of consciousness

The hallmark indications of CNS-OT are LOC and generalized tonic-clonic seizures^3^ that can last up to one to three minutes if P_I_O_2_ is immediately reduced by changeover to compressed air at onset of convulsions (Paul Bert Effect). Incontinence can also occur, and convulsions are said to resemble the major attack of idiopathic epilepsy [9,89,99]. Onset of seizures can invariably interrupt patient HBOT [161], induce cardiogenic pulmonary edema [82,84], and in the worst cases, result in death by drowning while diving [129]. Once convulsions cease, consciousness returns, but without memory of the event. Some individuals exhibit post-seizure symptoms that include nausea, vomiting, and impaired cognitive function lasting several hours (hangover), which is characterized by stupor, confusion, headache, and drowsiness [89,191]. Additional episodes of convulsions may occur if P_I_O_2_ is not immediately reduced with onset of first seizures, and may also recur during decompression on air [12,89]. Animal studies indicate that if P_I_O_2_ is not lowered during onset of the first set of convulsions, a second and third bout of seizures occurs separated by an interictal period of varying duration ranging from tens of seconds to tens of minutes [12,116]. In rodents, the initial event is often underwhelming and increases in intensity and duration during the subsequent episode of convulsions. Remarkably, the unanesthetized, freely behaving rat usually appears normal for all intents and purposes during the first interictal period [116]. Animal studies indicate that uninterrupted protracted exposure to HBO_2_ that produces recurring seizures ultimately causes death during status epilepticus [12,18]. Moreover, recurring exposures to an extreme level of HBO_2_ eventually induces irreversible hyperoxic paralysis [12,14], which was first described by Dr. John W. Bean (1901–1987) and is known as the “John Bean Effect” [9].

In addition, onset of O_2_-induced seizures occurs concomitantly with brainstem activation and massive catecholamine release and sympathetic outflow that depresses left ventricular function and subsequently increases arterial and pulmonary vascular pressure resulting in cardiogenic pulmonary edema [82,84]. The result of cardiogenic pulmonary edema is the same as pulmonary oxygen toxicity; that is, pulmonary edema. Pulmonary O_2_ toxicity, in contrast, results from diffuse redox and inflammatory damage to the pulmonary capillary endothelium and alveolar epithelium that impairs gas exchange and initiates neutrophil infiltration leading to respiratory failure [82,86]. Pulmonary O_2_ toxicity is also known as the “Lorraine Smith Effect”, in honor of the physiologist (Dr. James Lorraine Smith, 1862–1931) who first reported the malady [168]. Unlike cardiogenic pulmonary edema, which requires onset of seizures to be activated, pulmonary O_2_ toxicity occurs at lower levels of P_I_O_2_, including as low as 0.5 ATA at normobaric pressure, and takes hours to days to develop. Breathing a sub-lethal (without seizures) level of HBO_2_ (1.4 ATA), however, accelerates onset of early symptoms of pulmonary O_2_ toxicity in navy divers [166].

Acute cardiogenic pulmonary edema, therefore, is linked to CNS-OT [82], but the exact origin of central sympathetic outflow remains unknown [82,84]. One likely important region is the caudal Solitary Complex (cSC) in the dorsal medulla oblongata, which is comprised of the nucleus tractus solitarius (NTS) and dorsal motor nucleus of the vagus. The cSC is an important cardiorespiratory control region in the brainstem [70], and certain neurons in this area are exquisitely sensitive to hyperoxia and pro-oxidants, exhibiting depolarization and stimulation of firing rate [52,68,69,135,140] and increased reactive oxygen and nitrogen species (RONS) production [50,51,115,135,159]; see below, Fig. 2, Fig. 3.

#### 3.2.2 Non-convulsive signs and symptoms

Numerous non-convulsive S/Sx often precede unconsciousness and seizures and are considered part of the toxic indications of CNS-OT [[87], [88], [89],^191^]. Non-convulsive indications in human divers have also been categorized as “probable symptoms” and “definite symptoms” that precede convulsions [38,183]. Non-convulsive S/Sx can be remembered using the pneumonic VENTID-C [144] for the following toxic indications: Vision (blurred and tunnel vision), Ears (tinnitus), Nausea and/or vomiting, Twitching/Tingling in peripheral and facial muscles, Irritability (changes in mental status), and Dizziness. Any of the foregoing non-convulsive S/Sx may precede onset of tonic-clonic Convulsions. Seizures, however, can also occur without a detectable non-convulsive toxic indication [87]. Non-convulsive indications vary in severity, are sometimes difficult to identify, and do not all occur concurrently [9,38,87,191]. Certain indications are more common than others; for example, severe lip twitching is the predominate sign that often precedes seizures in humans [87,191]. Donald [89] reported that lip twitching transitions into convulsive movement of the lips followed by “generalized jactitations” (i.e., involuntary spasms of a muscle or muscle group) or, alternatively, full blown tonic-clonic convulsions. In these cases, upon regaining consciousness, the subject's last memory was experiencing severe lip twitching.

The characteristics of the non-convulsive S/Sx support the hypothesis that certain brainstem cranial nerve (CN) nuclei and their circuitry and cardiorespiratory control centers are activated during the latent period. In fact, the collective evidence indicates that the pre-seizure period during HBO_2_ exposure is a period of brainstem (“bulbo”) activation or bulbo-excitation. For example, visual auras, hallucinations, and disturbances (vision or “V” in VENTID-C) suggest involvement of brainstem CN nuclei controlling vision (CN II: Optic nerve) and eye movement (CN III, IV, VII: Oculomotor, Trochlear, Abducens nerves, and brainstem and midbrain nuclei). Auditory auras suggest involvement of the auditory receptors (ear or “E”) and activation of CN VIII (Vestibulocochlear nerve), and nausea (“N”) and vomiting also indicates stimulation of CN VIII plus the vomit center in the brainstem [30,43]. Twitching of facial muscles (“T”), including the lips, implies stimulation of CN VII (Facial nerve). Dizziness (“D”) or vertigo indicates, again, involvement of CN VIII (Vestibulocochlear nerve) and brainstem centers [43]. Finally, heart rate and respiratory abnormalities imply activation of brainstem control centers that regulate cardiopulmonary functions [69,70]; see below, *Physiology and pathophysiology of HBO*_*2*_ *exposure and CNS-OT*.

### 3.3 Latent period

Despite being toxic, the use of HBO_2_ in operational, military, and hyperbaric medicine becomes practical due to the initial latent period during which the individual is symptom free. Predicting the duration for safe HBO_2_ exposure, however, has proved difficult due to the variability between individuals in their sensitivity to HBO_2_, and the variability within the same individual on different days [[87], [88], [89],^191^]. This problem is further compounded by the fact that the risk for developing CNS-OT is accelerated by immersion in water and exercise; see below, *Risk factors for CNS-OT*. Hence, the risk of developing a toxic indication of CNS-OT (including non-convulsive S/Sx) is greater for diving operations with exercise as compared to that during HBOT while seated quietly inside a dry hyperbaric chamber. The difference in risk (dry < wet) and the highly conservative limits incorporated into O_2_ breathing protocols is why the incidence of patients suffering CNS-OT during HBOT is low; e.g., incidence of seizures in patients treated with HBOT ranges from 0.0024% [192] to 0.03% [108,153].

**Fig. 1:**
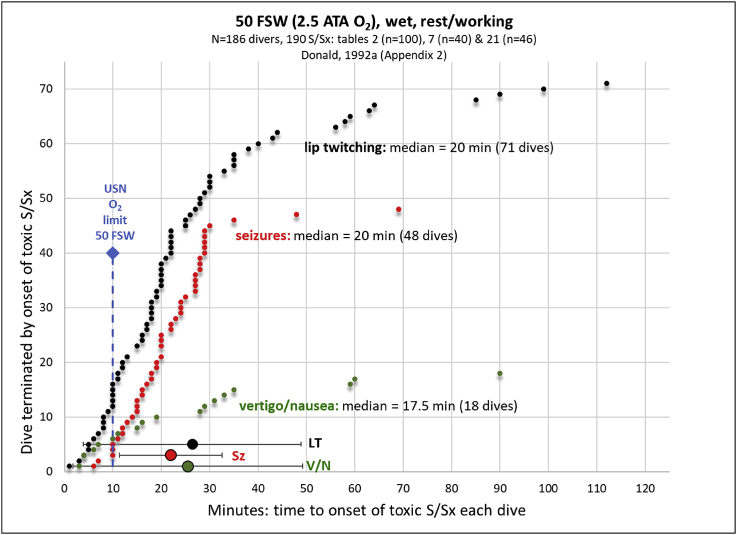
The effect of breathing 2.5 ATA O_2_ (50 fsw) while submerged either quietly or exercising on the latency to onset of either an early non-convulsive toxic end-point (lip twitching or vertigo/nausea) or seizures. Each data point represents a dive that was terminated early due to onset of an acute toxic indication of CNS-OT. Data are listed from shortest to longest latent period prior to onset of toxic end-point. Divers that did not develop any toxic indications are not represented. For comparison, the current USN limit for breathing HBO_2_ at 50 fsw (10 min) is indicated by the blue vertical, dashed line and diamond. In these dive trials, seizures (red symbols) occurred without any prior noticeable non-convulsive toxic indication. Experience showed that in cases when a non-convulsive S/Sx was missed or ignored that the diver developed seizures. Data are redrawn and average latencies are calculated by the authors using data sets in Appendix 2, Tables 2, 7, and 21 in Donald [87]. The maximum limit of the dive without an acute toxic indication was 30 min (Table 2: 100 dives, resting/working), ∼60 min (Table 7: 40 dives, resting) and 120 min (Table 21: 46 dives, working). LT, lip twitching (black symbols); Sz, seizures (red symbols); and V/N, vertigo/nausea (green symbols).

By contrast, Donald's wartime studies [[87], [88], [89]] revealed that when 100 divers were exposed to 50 fsw (wet, 2.5 ATA O_2_) while performing moderate exercise (a few rested quietly) for a maximum period of 30 min or until onset of an acute toxic end-point, whichever came first, that half of the divers (50%) developed acute toxic indications; 24 divers (24%) exhibited non-convulsive S/Sx first and another 26 divers (26%) developed seizures. The remaining 50 divers (50%) developed no toxic indications of CNS-OT before 30 min [88]. Fig. 1 summarizes data from this 100-man dive series (30 min maximum), plus two additional smaller dive trials (86 dives) that were all done at 50 fsw (2.5 ATA O_2_), submerged, and while either at rest or exercising up to a maximum exposure time of 60 or 120 min [86]. Pooling these three series of dives together reveals that 72% of dives were terminated early because O_2_ poisoning occurred, which is indicated in Fig. 1 by incidence of lip twitching (black symbols), nausea/vertigo (green symbols), and seizures (red symbols). Other non-convulsive S/Sx that occurred in four divers included throat spasms, abnormal respiration, body tremors, and headache/malaise (not shown). Based on these three series of O_2_-dives, the average latency ±SD to onset of an acute toxic end-point at 50 fsw was 26.4 ± 22.5 min (lip twitching), 25.5 ± 23.7 min (vertigo/nausea), and 22.0 ± 10.6 min (seizures). During the 30 min dive series (n = 100), divers either rested or performed mild exercise and their acute toxic indications began after 25.2 min ±6.9 min, range: 7–29 min. During the 120 min dive series (n = 40), all divers rested quietly, which extended the latent period, and acute toxic indications began after 40.7 min ±35.1 min, range: 3–112 min. Because of the variability observed in the latent period across a study population under wet conditions when pushed to acute toxic end-points [[39], [40], [41],[87], [88], [89],^183^,^191^], the current safe O_2_ exposure limits in the USN for breathing 100% O_2_ as a function of depth are highly conservative [127,144]; for example (depth (time at that depth, P_I_O_2_)): ≤25 fsw (240 min, 1.76 ATA O_2_), 30 fsw (80 min, 1.91 ATA O_2_), 35 fsw (25 min, 2.06 ATA O_2_), 40 fsw (15 min, 2.21 ATA O_2_), and 50 fsw (10 min, 2.5 ATA O_2_). Notice in Fig. 1 that the current USN exposure limit at 50 fsw is 10 min (blue diamond and dashed vertical line); however, most combat divers tolerated at least twice that dose of HBO_2_, and more in some divers, before developing acute toxic indications. The dose of hyperoxia is known as the oxygen concentration product, which is defined as the product of P_I_O_2_ and duration of HBO_2_ exposure.

**Fig. 2:**
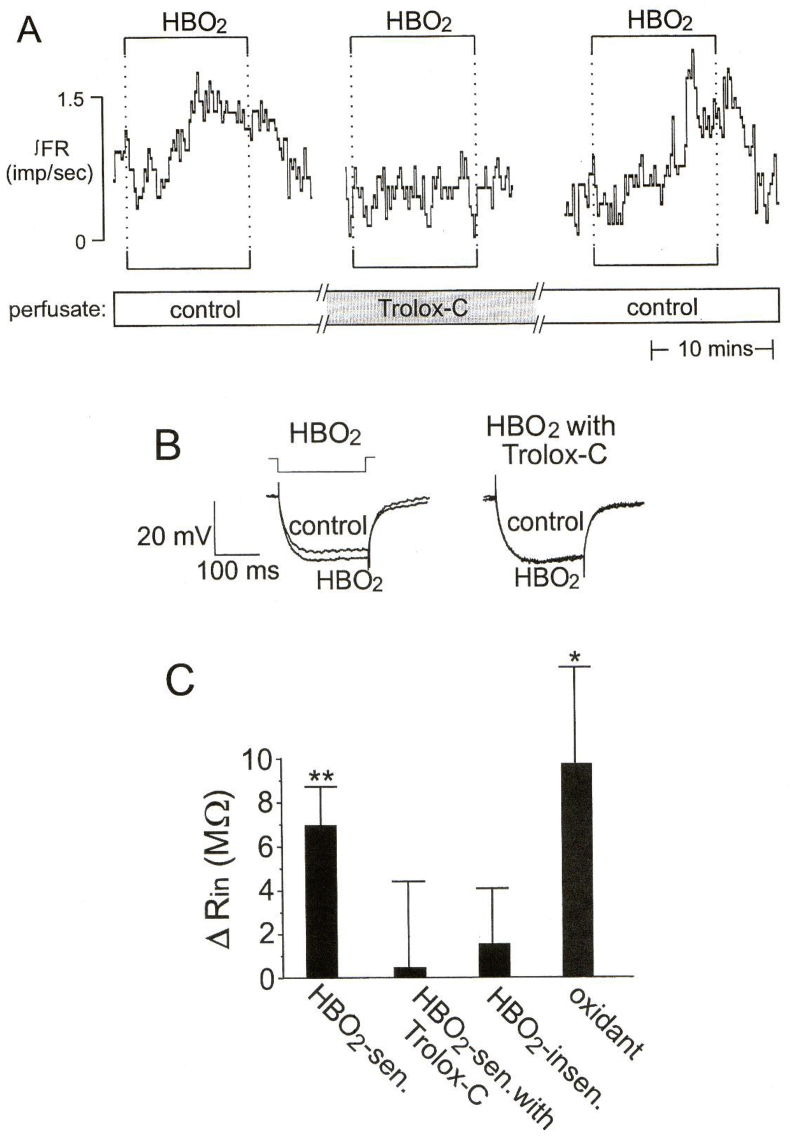
HBO_2_ increases input resistance, depolarizes membrane potential, and stimulates firing rate of neurons in the cSC in a rat brain slice. The excitatory effects of HBO_2_ are blocked by the antioxidant Trolox-C (100–200 μM), an analog of vitamin E. *A*) the trace of integrated firing rate (∫FR, impulses/s) measured via intracellular recording shows the ∫FR response to three bouts of HBO_2_ (3.3 ATA O_2_); control = 0.95 ATA O_2_ and P_B_ = 3 ATA helium. An initial exposure to 3.3 ATA HBO_2_ increased ∫FR. After 90 min of incubation in medium containing the antioxidant Trolox-C, a second exposure to 3.3 ATA HBO_2_ did not affect ∫FR. Washing out Trolox-C for 30 min restored the excitatory ∫FR response to a third exposure to HBO_2_. *B*) average membrane potential (V_m_) traces (n = 5) during −0.2 nA current injections show that the HBO_2_-induced increase in input resistance (R_in_; where R_in_ α 1/membrane conductance) also was blocked by Trolox-C. *C*) bar graph showing average increase (Δ) in R_in_ (means ± SE) of HBO_2_-sensitive (n = 31), HBO_2_-sensitive plus Trolox C (n = 4), and HBO_2_-insensitive (n = 43) neurons. **Values significantly different from zero (*t-*test) at P < 0.001. Figure reproduced with permission from Journal of Applied Physiology, Mulkey et al. [140].

**Fig. 3:**
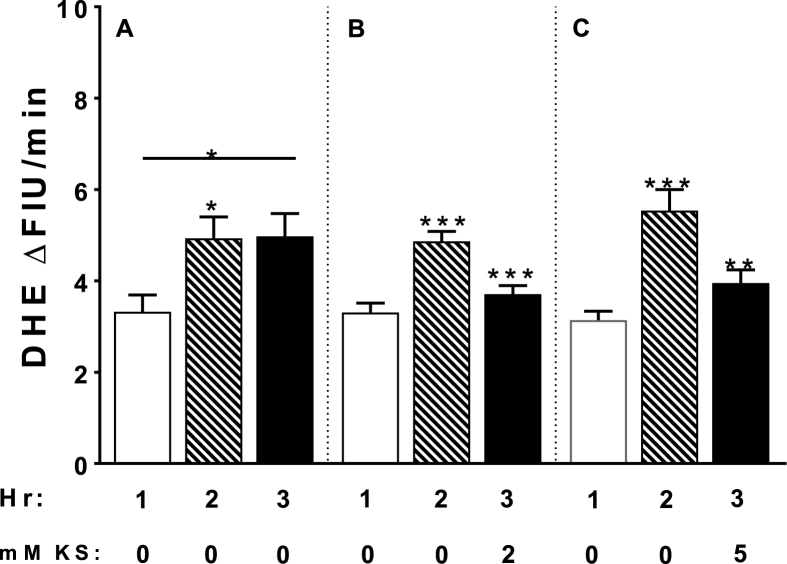
Effects of 0.95 ATA O_2_ on superoxide production in cSC cells in rat brain slices (300–400 μm thick) as measured by increased dihydroethidium (2.5 μM DHE) fluorescence over time. *A*) the rate of superoxide production significantly increased from hour 1 (control 0.40 ATA O_2_; open histogram) to hour 2 (hyperoxia 0.95 ATA O_2_; diagonal striped histogram) by 176% and remained unchanged throughout hour 3 of hyperoxia (black histogram). *B-C*) a 1:1 ratio of ketone salts (KS; β-hydroxybutyrate and acetoacetate) were added during the second hour of hyperoxia (hour 3; black histograms). Two and 5 mM KS significantly inhibited superoxide production by 20% (black histograms). Between 48 and 65 cells were analyzed in each experimental run (∼10 cells/brain slice/rat; postnatal age P10-39). Analysis of variance: *P < 0.05; **P < 0.01; ***P < 0.001. Unpublished data from Hinojo et al. [115].

### 3.4 Physiology and pathophysiology of HBO_2_ exposure and CNS-OT

Studies in anesthetized and unanesthetized instrumented animals^4^ reveal a predictable pattern of physiological changes over the course of the initial latent period and the ensuing bulbo-excitation period that implies increased neural excitability in autonomic and brainstem control centers prior to seizure genesis. Exposure to HBO_2_ initially results in a transient parasympathetic response comprised of decreases in heart rate, cardiac output, blood pressure, and sympathetic tone [80,100]. Initially, cerebral blood flow (CBF) is decreased by vasoconstriction and delays a significant increase in brain tissue PO_2_ [8,71,72,75,76,79]. Likewise, minute ventilation decreases initially due to hyperoxic inactivation of peripheral chemoreceptors [69,152]. With extended exposure to HBO_2_, this cardiopulmonary response is overtaken by increased sympathetic outflow resulting in hyperventilation and hypertension [98,100,152]. Part of the mechanism for the shift in autonomic output is compensation for pressure-induced activation of the arterial baroreceptor response [78]. Additionally, cooling of core body temperature has been reported following respiratory responses during HBO_2_ [38,93,155,165], possibly implicating fast-neural autonomic input from respiratory and thermosensitive centers in the brainstem into areas of temperature regulation in the hypothalamus [31,118,130,171]. In parallel with the sympathetic response, a buildup in nitric oxide causes escape from O_2_-induced cerebral vasoconstriction and CBF increases causing a surge in neural tissue PO_2_ and RONS production and, shortly thereafter, seizures [71,74,82]. Not all physiological changes, however, are easily measured in real time, especially under conditions of undersea operations. Regardless, recent and ongoing research is testing the effectiveness of certain key physiological responses as predictors of an impending seizure; that is, so-called “physio-markers” for early warning of a toxic CNS-OT hit. Potential physio-markers include hyperoxic hyperpnea/hyperventilation [69,152], heart rate and pattern [122,158], hyperoxic hypothermia [93,155,165], and possibly other predictors [20,86,191].

### 3.5 Risk factors for CNS-OT

The risk of developing an acute toxic indication of CNS-OT is increased by several conditions. Systemic CO_2_ retention commonly occurs in divers breathing HBO_2_ and shortens the latent period prior to seizure initiation [3,104,173]. While diving, CO_2_ production increases from exertion underwater, in conjunction with increased CO_2_ retention that results from the following factors: increased work of breathing due to increased density of inspired gases and thus increased airway resistance, and hydrostatic compression of the chest wall during respiration [68]; and increased dead space ventilation caused by the additional length of plumbing added to the diver's conducting zone “airway” by the underwater breathing apparatus [107]. Initially, breathing a hyperoxic gas mixture also inhibits pulmonary ventilation, but this is short-lived and continued exposure to hyperoxia stimulates ventilation, which counteracts CO_2_ retention as long as the diver is not rebreathing CO_2_ [69,152]. The net effect of all the foregoing is increased CO_2_ production and CO_2_ retention to increase the partial pressure of CO_2_ (PCO_2_) in the blood producing respiratory acidosis. The situation is exacerbated by the O_2_-induced rightward shift in the CO_2_ transport curve (Haldane Effect) producing a higher dissolved PCO_2_ in the blood at the expense of bicarbonate and carbamino compounds [15]. The net effect of systemic hypercapnic acidosis is to further enhance RONS production for a given level of P_I_O_2_ as follows: hypercapnia increases CBF and thus brain tissue PO_2_ [104,120], and CO_2_ reacts with peroxynitrite (produced from O_2_-induced superoxide and nitric oxide [50]) and protons free up iron from transferrin to enhance the Fenton Reaction [51,66]. Together, RONS production increases more so than under normocapnic conditions, which presumably accelerates the oxidative injury producing CNS-OT; see below, *Free radical production and redox stress.*

Additional important risk factors that accelerate onset of CNS-OT are exercise, immersion, and cold [20,86,191]. As stated, exercise increases end-tidal CO_2_ with the consequences just listed above. Immersion is a critical risk factor, particularly in diving as limits for dry HBO_2_ exposure (i.e., HBOT, recompression therapy) are reduced from 2.4 ATA to only 1.3 ATA in wet conditions. For example, a series of human diving experiments using 100% O_2_ resulted in 3/6 participants completing a 2-h dry dive with no symptoms, while 6/6 participants experienced CNS-OT symptoms during immersion at the same depth [87]. While the mechanism/s underlying this phenomenon are unknown, it is suggested that immersion contributes to CO_2_ retention by compounding airway resistance and work of breathing, as well as increasing cardiac output due to compression-induced diuresis. Likewise, cold temperatures reduce blood flow to the skin and periphery to conserve core temperature, resulting in increased venous return and cardiac output with subsequent redistribution of blood volume to the CNS and thus increased O_2_ delivery [150]. Conversely, acclimatization to heat has been shown to significantly increase seizure latency, presumably via upregulation of heat shock protein 72 that coincided with decreased CO_2_ production [6].

Finally, other factors modulating the risk for CNS-OT include sleep status and circadian rhythm [85], inert gases [4,22,34], diet [27], and gender [111,167]. For example, rodent studies have shown significantly decreased seizure latency in females versus their male counterparts [111]. Likewise, the incidence of CNS-OT was higher in female divers (4.4% acute toxic indications with 1.3% seizures) versus males divers (1.4% acute toxic indications with 0.4% seizures) that were treated by HBOT for dysbarism [167]. The higher risk for CNS-OT in females may possibly be due to estrogen's proconvulsant properties that result from estradiol (E2)-augmented N-methyl-d-aspartate (NMDA)-mediated glutamatergic receptor activity. Additionally, E2 has been shown to increase the excitability of CA1 hippocampal neurons via upregulation of NMDA receptors and inhibition of Gamma Aminobutyric Acid (GABA)-ergic neurons. Conversely, low estrogen levels have been shown to decrease the number of pentylenetetrazole-induced seizures, as well as increasing latency to said seizures [186].

## 4 Current theories and hypotheses on neural mechanisms of CNS-OT

The technical challenges of remotely making neurophysiological measurements in real time from anesthetized instrumented animals and reduced brain tissue preparations that are sealed inside a pressurized hyperbaric chamber, while the investigator remains at room pressure, plus the rigorous safety requirements for using electrical equipment (e.g., motorized micromanipulators, stimulating microelectrodes, electrophysiology preamplifiers, respiratory pumps, syringe pumps, thermoregulation equipment, etc.) in combination with the highly flammable environment created by pressurized oxygen [163] have, not surprisingly, impeded research on the neural mechanisms of CNS-OT. Adapting cutting edge research tools for safe use under hyperbaric conditions and hyperoxia requires extra motivation, persistence, and a bit of creativity. Having said that, progress has been achieved, which is summarized here. Importantly, CNS-OT seizures resemble convulsions of idiopathic epilepsy [9,89,100]. Accordingly, based on theories borrowed from research on epilepsy under normoxic conditions, plus the results from animal physiology experiments (in vivo and in vitro) that describe the central effects of HBO_2_, we propose that HBO_2_-seizures develop in subcortical regions (oxtox trigger zones) and progressively spread throughout two looping and reverberating-amplifying circuits in the brainstem and cerebral cortex, as outlined in the following section.

### 4.1 Seizure genesis and propagation

Typically, tonic-clonic seizures are evoked from trigger zones, specific loci in the CNS with low thresholds for seizure initiation in animal models when irritated by focal application of convulsant chemicals; e.g., focal application of kainic acid into the CA3 hippocampal cortex [33] or bicuculline into the area tempestas of the piriform cortex [36] evokes tonic-clonic seizures in rodents. Likewise, we propose that there are oxtox trigger zones in subcortical nuclei containing neurons and local circuits with low thresholds for activation during exposure to hyperoxia. Our working hypothesis is that neuronal sensitivity to hyperoxia is evidence that redox and nitrosative signaling mechanisms have a significant role in the various functions ascribed to these neurons under normoxic conditions [66,90,140]. For example, medullary centers controlling cardiorespiration and digestion, such as the cSC of the dorsocaudal medulla oblongata, an O_2_-sensitive region [69] discussed above in the context of acute cardiogenic pulmonary edema, are activated by natural stimuli such as arterial/interstitial/intracellular PCO_2_ and pH, arterial pressure, airway stretch and irritation, intragastric pressure, upper esophageal stretch, orexin, etc [67,70]. Under normoxic conditions, the various redox and nitrosative signaling mechanisms employed in these functions depend in part on rates of production and inactivation of various RONS, which are fine-tuned by the complement of extracellular, intracellular, and mitochondrial oxidative enzymes (e.g., nicotinamide adenine dinucleotide phosphate (NADPH) oxidase, superoxide dismutase, nitric oxide synthase), free metals (e.g., iron), antioxidants, and the functional energetics of the mitochondrial respiratory chain [179], and normal variations in regional tissue PO_2_ [68]. During early exposure to HBO_2_, however, neurons and local circuits endowed with significant redox signaling mechanisms are activated by the unnatural stimulus of a hyperoxic atmosphere, which causes focal depolarization and hyperexcitability. Nuclei activated early on function as oxtox trigger nuclei and produce high frequency patterns of action potentials; i.e., firing rate [52,68,96,97,140]. Importantly, not all neurons are stimulated by HBO_2_ [135,140]. For example, Fig. 2A–C shows an intracellular recording of a cSC neuron in a rat brain slice that increased firing rate and input resistance during exposure to HBO_2_. The effects of HBO_2_ firing rate and input resistance were blocked by the antioxidant Trolox-C [140]. These data suggest that HBO_2_ stimulates certain cSC neurons by decreasing an outward conductance, presumably potassium, in response to cellular oxidation [52,140].

Based on research using models of epilepsy [36,37,45,121,189], our theory predicts that neural activity originating in oxtox trigger zones is quickly propagated along axons and across chemical synapses [96,97,125] to adjoining neurons arranged in oscillatory, looping-amplifying circuits that otherwise function normally during normoxia in learning and memory consolidation/recall, and basic emotions and drives (feeding, sex, etc.). Under unnatural, neuropathological conditions (e.g., drug-induced seizure genesis, idiopathic epilepsy, and HBO_2_ and redox stress), hyperexcitability and hypersynchrony of neural electrical activity in these same looping circuits produces generalized tonic-clonic motor seizures [45,189]. Currently, two mostly independent reverberating, looping-amplifying circuits are thought to exist in the mammalian CNS that produce generalized seizures. One looping circuit resides in the forebrain (cerebral cortex, limbic system, and basal ganglia) and is responsible for expression of clonic seizures, facial and forelimb clonus, and rearing and falling over. For example, in the CA1 hippocampus, which is part of the limbic system, a single bout of hyperoxia, including reoxygenation following hypoxia or exposure to HBO_2_ applied over several minutes induces long-lasting stimulation of bursting activity (at least 45 min) in CA1 pyramidal neurons subsequent to Schaffer collateral stimulation; that is, a sustained increase in excitatory post-synaptic neurotransmission known as oxygen-induced potentiation or OxIP [96,97]. Such an O_2_-induced phenomenon could conceivably contribute to amplified neural excitability following onset of exposure to HBO_2_. In addition to this forebrain circuit, a second neural loop resides in the brainstem (reticular formation of the medulla oblongata, pons, and midbrain, and specific nuclei of the hypothalamus and thalamus) and is responsible for the expression of tonic convulsive seizures and running/bouncing clonic convulsions [36,37,121]. Nodes of communication between the two circuits enable communication and recruitment of new brain areas into the ictal network to cause more complex seizures [36,94]. The variety of motor behaviors exhibited immediately before and during HBO_2_-seizures in rodents may depend on the degree of coordination between these two circuits [116].

What is the evidence that HBO_2_-seizures develop first in subcortical regions, and progressively spread throughout “reverberating, looping-amplifying circuits” in the brainstem and on to the cerebral cortex? Investigators have searched for sites of seizure genesis in various animal models exposed to HBO_2_ while measuring regional neural activity using multi-electrode recordings. Neural activity increases in the forebrain and subcortical areas, including the thalamus, hypothalamus, basal ganglia, and brainstem; however, no consistent temporal relationships between different regions have been established before and during electrical convulsions [19,28,59,110,160,169]. Some investigators proposed that HBO_2_-seizures originate in the cerebral cortex [5,59] and/or cerebellum [169]. For example, some investigators report that a first electrical discharge occurs in the cerebral cortex several mintues before onset of behavioral (motor) seizures [5]. The initiation of seizures in the cerebral cortex is unlikely, however, for two reasons. First, cortical electroencephalogram activity doesn't always increase prior to manifestation of behavioral seizures [116]. Second, removal of the forebrain [13,160] or bisecting the corpus callosum [103] does not abolish seizures during HBO_2_. A medullary pyramidotomy, however, which severs the corticospinal tract, abolishes seizures during HBO_2_ [11,110]. Thus, the few lesioning studies to date suggest the critical region for seizure initiation during HBO_2_ lies below the cerebral cortex [13,103,160] and above medullary pyramids [11,110].

Further evidence for a subcortical site of seizure genesis during exposure to HBO_2_ is seen with measurements of regional CBF in rodents during hyperoxic exposure. Seizures are preceded by cerebral vasodilation, which increases CBF, neural tissue PO_2_, and RONS production [76,79]. Consequently, regions of the CNS where the earliest and largest increases in CBF occur are thought to be important in seizure genesis. Gasier et al. [98] reported higher blood flow and tissue PO_2_s in subcortical areas, followed by the cerebral cortex and cerebellum, but with no changes in CBF seen in the substantia nigra (part of the basal ganglia). Blood flow increased earliest in the striatum (another part of the basal ganglia); however, micro-dialysis of nitric oxide synthase inhibitors into the striatum did not alter the latency to seizure. Other subcortical areas that exhibited marked increases in local blood flow included the hippocampal cortex (limbic system), hypothalamus (diencephalon), and NTS (medulla oblongata).

Of the regions identified above using multi-electrode recordings and regional CBF measurements during HBO_2_, only the cSC (medulla oblongata) and CA1 hippocampus (limbic system) have been studied using single cell electrophysiology under HBO_2_ conditions, and both regions were found to contain neurons that are stimulated during hyperoxia [52,96,97,125,140]; e.g., Fig. 2 (cSC). Likewise, both regions increase RONS production during hyperoxia [50,51,64,115,135,159]; e.g., Fig. 3 (cSC). Regarding the NTS, which is part of the cSC, it is an area of central CO_2_ chemoreception and cardiorespiratory control [70]. The NTS also receives afferents from arterial baroreceptors [55], the hippocampus [44], and the hypothalamus [56], all regions that increase regional CBF prior to seizure genesis [98]. Therefore, based on the foregoing data, we propose that the cSC, at a minimum, is involved in the various autonomic and cardiorespiratory responses observed during the bulbo-excitation period that precedes seizures during exposure to HBO_2_ (*Physiology and pathophysiology of HBO*_*2*_ *exposure and CNS-OT*). Moreover, we propose that the HBO_2_-sensitive properties of the cSC described herein make it a candidate as a potential oxtox trigger zone. In summary, lesioning studies and measurements of regional CBF support the idea that possible sites of seizure genesis reside in the brainstem (midbrain, pons, and medulla oblongata); however, given the paucity of studies that have tested the sensitivity of neurons and networks during HBO_2_ (in vitro and in vivo), we anticipate that neurons in other subcortical nuclei, in addition to the cSC, will be identified as potential oxtox trigger nuclei in future studies.

### 4.2 Neuronal sensitivity to pressure per se, gas partial pressure, and oxidation

Breathing a hyperbaric gas mixture, whether it is pure O_2_ or an O_2_-enriched mixture containing inert gases (e.g., nitrogen and/or helium), has three actions on brain tissue. First, the partial pressure of each gas species in the breathing gas mixture affects neuronal activity in a way that is determined by the gas' molecular weight and lipid solubility in the plasma membrane [62]. In the case of molecular O_2_, narcotic actions have been reported [112], but they are difficult to determine given the high reactivity of molecular O_2_ to form singlet oxygen, superoxide and nitric oxide [50,52,64,115,159], and its consumption by mitochondrial respiration [128,179].

The second action on the brain of breathing a hyperbaric gas mixture is the influence of increased ambient pressure surrounding the diver or patient. At the systems level, a pressure-applied force against the surface of the body immediately equilibrates throughout extracellular/intracellular fluids of neural tissues. The range of hydrostatic pressures at which toxic indications of CNS-OT occur are too small for any thermodynamic and kinetic effects to be of physiological significance [68]. Alternatively, at the cellular level, it was postulated that the various non-fluid nanostructures comprising the plasma membrane, ion channels, and cytoskeleton undergo differential rates of compression during pressurization at levels relevant to CNS-OT. This is hypothesized to produce localized shear and strain forces between adjoining nanostructures that perturb neurotransmitter release and ion channel gating [132]. Certain neurons in the cSC exhibit barosensitivity and HBO_2_-sensitivity. Neuronal barosensitivity and HBO_2_-sensitivity in the cSC, however, are not well correlated in the same neuron. Moreover, both stimuli depolarize and increase firing rate in certain cSC neurons, but by different membrane mechanisms; HBO_2_ decreases membrane conductance and pressure increases membrane conductance [141]. Thus, at least in cSC neurons, pressure per se does not appear to be a significant factor in mediating cellular O_2_-sensitivity during exposure to HBO_2_.

Finally, the third action of breathing hyperbaric gas mixture on the brain is the ability of gas molecules to undergo secondary chemical reactions that yield biologically reactive byproducts. This is generally regarded as the primary mechanism by which molecular O_2_ mediates, first, its stimulatory actions on the CNS (e.g., oxtox trigger neurons, bulbo-excitation), and ultimately, its toxic effects culminating in seizures. Unfortunately, as stated above, there have been relatively few studies on this issue due to the technical challenges of working at hyperbaric pressure and hyperoxia. This technical challenge is compounded by the fact that nearly every active neuroscientist uses hyperoxic “control O_2_” conditions when studying neuronal function in reduced tissue preparations under normobaric conditions; that is, 95% O_2_-balance CO_2_ in a bicarbonate buffered medium, or 100% O_2_ in a HEPES-buffered medium [68,139]. We have discovered that 0.95 ATA “control O_2_” produces a range of tissue PO_2_ in a 300–400 μm thick brain slice that is equivalent to an intact animal breathing 2.0–2.5 ATA O_2_; i.e., HBO_2_ minus the effects of pressure per se [68,96,139]. Moreover, four hours of exposure to 0.95 ATA O_2_ increases cell death in hippocampal brain slices compared to lower levels of oxygenation; e.g., 0.4 ATA O_2_ [64]. We would propose, therefore, that the widespread use of 95–100% “control O_2_” in an avascular, diffusion dependent, reduced cell/tissue preparation of the mammalian CNS is really a model of chronic exposure (hours) to hyperoxia in which the mechanism under study is impacted or modulated by protracted activation of any unrecognized redox signaling mechanisms present, irrespective of whether those redox signaling mechanisms were the original intent of the study. More recently, we have decreased the level of control O_2_ used in our rat brain tissue slice studies from 0.95 to 0.4 ATA O_2_ and have revisited or replicated many of our earlier findings regarding O_2_- and CO_2_-sensitivity of cSC neurons when using 0.95 ATA “control O_2_”; e.g., under these new control O_2_ conditions, 0.95 ATA stimulates firing rate and RONS production as does HBO_2_ [[50], [51], [52],^115^,^135^,^159^]. Continued research will likely show the mechanism/s underlying cellular hyperexcitability during HBO_2_ will include alteration of neurotransmitter levels (see below, *GABA:Glutamate ratio*), as well as direct and/or indirect effects on cellular equilibrium via oxidation of redox sensitive ion channels or physical alteration to the plasma membrane [62].

### 4.3 Free radical production and redox stress

Hyperoxia is a key factor in the production of two primary RONS: superoxide and nitric oxide. Superoxide is created via several sources, most notably electron leak from Complexes I and III in the electron transport chain, as well as NADPH oxidase, xanthine oxidase, and uncoupled nitric oxide synthase [90,105,178]. Additionally, a third primary RONS, singlet oxygen, can be produced by a combination of hyperoxia and inert gas exposure at pressure [113,136,159,174]. This may explain why O_2_-enriched inert gas mixtures enhance CNS-OT in animals [4,22,34] and redox stress in single-cell organisms [175,176].

Despite its relatively low reactivity, superoxide has very fast reactions with the enzyme superoxide dismutase in the formation of hydrogen peroxide and nitric oxide in the formation of peroxynitrite. Measurement of superoxide in mammalian cells is easily accomplished with the use of fluorometric dyes, such as the superoxide-specific dye Dihydroethidium (DHE) [190]. DHE has been used in several studies testing the direct effects of hyperoxia on superoxide production in acute (rat) brain slices, including neurons in the CA1 hippocampus [64] and cSC [50,115]. In these studies, superoxide was shown to increase as a function of O_2_ over a broad range of normobaric and hyperbaric tissue slice PO_2_s. However, measurement of increased superoxide levels in the cSC required the use of a cocktail of nitric oxide synthase and superoxide dismutase inhibitors to allow for adequate binding of superoxide to DHE.

Nitric oxide, while involved in the production of peroxynitrite via its fast reaction with superoxide, also plays a critical role as a potent vasodilator [35,74]. Like superoxide, nitric oxide has also been shown to increase during hyperoxia in cSC neurons using the fluorogenic dye 4-Amino-5-Methylamino-2′,7′-Difluorofluorescein Diacetate, again requiring the use of a pharmacological cocktail, in this case superoxide dismutase mimetics, to measure increase nitric oxide production during hyperoxia [50]. Measurement of cerebral nitric oxide in rodents exposed to HBO_2_ have shown that nitric oxide levels are initially kept low by concurrent superoxide production. Studies by Demchenko et al. [72] have revealed superoxide is in fact protective with respect to CNS-OT seizures, with overexpression and inhibition of superoxide dismutase resulting in decreased and prolonged seizure latencies, respectively. As HBO_2_ exposure becomes prolonged, nitric oxide levels begin to overrun those of superoxide, reaching a breakpoint that results in a sharp increase in both cerebral nitric oxide and subsequent vasodilation that are followed by seizure onset shortly thereafter [71,76,81]. Interestingly, repeated seizure activity has been shown to decrease latency to seizure onset during recurring exposure to HBO_2_, in part due to upregulation of both superoxide dismutase and nitric oxide synthase [49]. As expected, inhibition of nitric oxide synthase via several pharmacological agents significantly delays seizure onset during exposure to HBO_2_ by prolonging cerebral vasoconstriction during HBO_2_ and preventing nitric oxide-induced cerebral vasodilation and delivery of hyper-oxygenated blood to the brain that ultimately precipitates further RONS production and seizures [21,49,76]. Likewise, other agents that induce vasoconstriction, such as caffeine, have shown efficacy as well in delaying seizures [26].

Carbon dioxide is an important risk factor in the onset of CNS-OT due to its effects on minute ventilation and O_2_ uptake, as well as cerebral vasodilation and O_2_ delivery to the brain [144]. As expected, the addition of CO_2_ to a hyperoxic breathing gas mixture significantly increases cerebral PO_2_ compared to hyperoxia alone [120]. Importantly, CO_2_ also has a critical, biologically relevant reaction with peroxynitrite, forming the intermediate nitrosoperoxocarboxylate that spontaneously yields the nitrogen dioxide radical and carbonate radical, which are powerful nitrating and oxidizing agents, respectively [66]. Increased PCO_2_ concurrently results in proton formation, either by mass action or enzymatically via carbonic anhydrase. The resulting acidosis stimulates the Fenton Reaction, which converts hydrogen peroxide into the highly reactive hydroxyl radical. These reactions have been reviewed and summarized by Dean [66] and tested in cSC neurons, the results of which revealed that hyperoxia is able to increase these downstream RONS and, moreover, their production is exacerbated by the addition of hypercapnic acidosis [52]. Interestingly, despite the potential for these secondary RONS to cause widespread cellular damage, little evidence was seen in these same cSC-containing brainstem slices after four hours of hyperoxia (2 ATA O_2_), possibly due to upregulation of antioxidant defense mechanisms or degradation of damaged lipids and proteins [52].

Not unexpectedly, the risk for CNS-OT increases when these hyperoxia-dependent RONS are overproduced and overwhelm endogenous antioxidant defense mechanisms. Studies measuring RONS production and markers of redox and nitrosative stress have noted that both increase in the brains of animals exposed to HBO_2_ [71,77,99,147,151]. RONS, such as nitric oxide, hydrogen peroxide, and hydroxyl radicals have been shown to increase prior to CNS-OT seizure induction [71,91,177,193], while byproducts of protein nitration and oxidation (3-nitrotyrosine and carbonyl groups, respectively) are seen both prior to and following seizure onset [46]. Thus, treatment with an array of antioxidants, such as nitric oxide synthase inhibitors [21,194], catalase [114], beta-carotene [23], and superoxide dismutase mimetics [24,25] have significantly prolonged seizure onset.

### 4.4 GABA:Glutamate ratio

Animals exposed to HBO_2_ exhibit decreased ratios of the inhibitory neurotransmitter GABA to the excitatory neurotransmitter glutamate (Glu), primarily due to decreased levels of GABA relative to an unchanged level of Glu [73]. This decrease in GABA can be attributed, either wholly or in part, to nitrosylation of GAD65 [99], which converts Glu to GABA. Indeed, treatment with NOS inhibitors partially rescues this detrimental change in the ratio of neurotransmitters [73]. Likewise, seizure latency during HBO_2_ has been extended with excitatory amino acid antagonists [60]. The excitatory effects of hyperoxia listed above have been mimicked by exposure of neuronal tissue to the chemical oxidants Chloramine-T and N-chlorosuccinimide, which exert their effects via oxidation of Methionine and Cysteine residues [140,142]. Importantly, the stimulatory effects of chemical oxidants on neuronal hyperexcitability were not always reversible. Increasing evidence suggests some ion channels are redox regulated, with oxidation by RONS and fast reduction by enzymes (e.g., thioredoxin, methionine sulfoxide reductase) and antioxidants (e.g., glutathione) [53,54,61,123,124,162,172]. While it remains unknown whether CNS-OT seizures are the result of redox stress, the rapid cessation of seizure activity following the removal of hyperoxic gas mixture suggests the cause is more likely the result of abnormal activation of redox signaling mechanisms.

## 5 Mitigation strategies of CNS-OT

As illustrated in Fig. 1, the current limits on HBO_2_ breathing are conservative. The USN's goal is to expand the envelope of clandestine diving operations; that is, longer and safer dives. Currently, the only approved strategy for prevention of CNS-OT is reduction of depth (chamber pressure), and thus, P_I_O_2_. There are, however, additional experimental mitigation strategies that have produced promising results with respect to increasing latency time to seizure onset in animal models that include the following: antiepileptic drug therapy (AEDs), anti-adrenergic drug therapy, ketone metabolic therapy, and hyperbaric oxidative preconditioning (HBO_2_-PC). The most promising AEDs tested to date fall within the categories of sodium-channel antagonists and, not surprisingly, GABA reuptake inhibitors [83,106,180]. In addition to affecting electrochemical equilibrium and GABA levels in the brain, AEDs may also exhibit antioxidant properties [134,148,149]. Likewise, anti-adrenergic therapy has been shown to be highly efficacious, particularly with non-specific blockade of α_1_/α_2_ and β_1_/β_2_ receptors [100]. However, treatment with AEDs and anti-adrenergic medications can result in cognitive and physical deficits, with symptoms including drowsiness, dizziness, weakness, nausea, vomiting, and diarrhea [119,137].

The ketogenic diet has also long been utilized for seizure regulation [187,188]. However, the ketogenic diet is a relatively restrictive diet, making a state of ketosis difficult to maintain in patient populations and military personnel. Thus, exogenous ketone esters, which induce a state of therapeutic ketosis by elevating blood ketone bodies within 30 min of oral delivery, and without dietary alterations, have been shown to significantly increase the latency time preceding seizure to a similar degree as AEDs and other therapies [2,63]. Ongoing animal behavior trials in the authors’ laboratory indicate that exogenous ketone metabolic therapy does not impair animal performance (unpublished findings). Importantly, ketogenic supplementation requires the elevation of the ketone bodies acetoacetate and acetone, and not just β-hydroxybutyrate [47,63]. Unlike AEDs, the neuroprotective mechanism allowed for by ketones is unknown, but most likely involves, at least in part, decreased RONS production. Fig. 3A–C shows the effects of ketone salts on O_2_-induced superoxide production in cSC cells in a rat brain slice [115]. Exposure to hyperoxia during hours 2 and 3 increases superoxide production consistently (3A). Addition of ketone salts during hour 3 at a concentration of 2 mM (3B) and 5 mM (3C) significantly inhibited superoxide production during exposure to hyperoxia. Inhibition of RONS production during ketosis may include stabilization of the electron transport chain, antioxidant effects, alteration of inhibitory neurotransmitter levels, or a combination of these factors and others [115,131]. As with other therapeutic options, exogenous ketone supplementation has side effects, which can include hypoglycemia and gastrointestinal distress [92,185].

It is well known that systematic interruptions in exposure to hyperoxic gas mixtures using air over time, otherwise known as intermittent hyperoxia, extends tolerance of the brain and lung for oxygen toxicity [48,57,126]. Neuroprotection against oxidative stress has been demonstrated in rodents using non-convulsive levels of HBO_2_-PC prior to exposure to a greater lethal level of HBO_2_ that induces seizures. In this case, HBO_2_-PC increased the latency period before seizure [7]. The critical step in initiating protection is a non-lethal increase in RONS induced by intermittent exposure to either HBO_2_, ischemic hypoxia and reoxygenation, or hypoxic hypoxia and reoxygenation [95,156,157]. Regarding HBO_2_-PC, the fact that exposure to 2.0 ATA did not induce neuroprotection, but 2.5 ATA did, suggests a threshold level of RONS production is required to activate the mechanism [95]. Oxidative preconditioning involves de novo protein synthesis since its protective effects against oxidative stress are blocked by cyclohexamide and actinomycin-D [10,170]. Important proteins activated by increased RONS during oxidative preconditioning include several important antioxidant enzymes such as catalase, superoxide dismutase, and glutathione peroxidase [109,146]. Administering an antioxidant to the animal during preconditioning will significantly decrease the level of neuroprotection that develops, further supporting the importance of RONS in induction of O_2_-tolerance [157]. The increased activity of antioxidant enzymes following preconditioning suggests that RONS produced during subsequent periods of oxidative stress are buffered by the increased antioxidant defenses. Additionally, it has been suggested that preconditioning induces substrate limitation in the mitochondria thereby slowing down the Krebs Cycle, reducing electron flow through the respiratory chain, and producing less RONS [95].

## 6 Conclusions and recommendations for future research on CNS-OT

CNS-OT is a complex syndrome that presents as a variety of non-convulsive S/Sx, as well as generalized tonic-clonic seizures with LOC. While CNS-OT is not deadly in and of itself, the conditions under which HBO_2_ is used in hyperbaric and undersea medicine can result in injury or even death if P_I_O_2_ remains elevated after toxic indications appear; for example, suffering generalized tonic-clonic convulsions while recovering from surgery (HBOT) or underwater (diving). Whereas the dangers associated with LOC and seizures are self-evident, certain non-convulsive S/Sx are also potentially incapacitating (e.g., nausea, vomiting, vertigo, dizziness, and cognitive impairment), especially during HBOT following surgery, during clandestine diving operations, or when preparing for escape or rescue during a pressurized DISSUB emergency. Consequently, the hyperbaric and undersea medical research communities are motivated to develop strategies for predicting and delaying a toxic hit during HBO_2_ exposure. The challenge in extending the latent period and accurately predicting an impending toxic indication, however, is the variability in sensitivity within a population to HBO_2_ and a given person on different days, for reasons yet to be discovered. Research to date suggests that seizures originate in subcortical nuclei and spread to higher cortical centers. Research also confirms an important role for brainstem autonomic and cardiorespiratory centers in the pathogenesis of CNS-OT and acute cardiogenic pulmonary edema. Important fundamental questions remain, however, such as the site/s of seizure genesis and the underlying molecular, cellular, and neurochemical mechanisms that produce CNS hyperexcitability. Regarding the underlying mechanisms, clearly RONS are involved in terms of regulating CBF, depolarizing neurons, and increasing overall excitation by reducing inhibitory synaptic neurotransmission. Importantly, future research on the underlying molecular and cellular mechanisms needs to focus on defining appropriate levels of both control oxygenation and hyperoxygenation for neurons and circuits as additional regions of the CNS are investigated, such as subcortical nuclei that are implicated in seizure genesis. Additionally, a potentially useful strategy for extending the currently conservative O_2_ exposure periods includes identifying, modeling, and testing the reliability of “physio-markers” to predict an impending toxic HBO_2_ hit. Several possible mitigation strategies have also been identified that delay seizures, including ketogenic metabolic therapy, AED therapy, anti-adrenergic drug therapy, and HBO_2_-PC; however, testing for possible adverse side effects of these treatments will be required. For example, what are the long-term effects on CNS health and function and cognition that might negatively impact diver performance or patient wellbeing? If a certain mitigation strategy extends CNS tolerance to HBO_2_, are there adverse consequences such as increased risk of pulmonary oxygen toxicity despite a lengthened CNS latent period? Finally, beyond solving an important medical problem in hyperbaric and undersea medicine, HBO_2_ animal models of CNS-OT (in vitro and in vivo) provide useful models for studying the central effects of oxidative stress in disease and, additionally, provides an alternative model for research on seizure genesis in the context of epilepsy.

## Disclaimer

The views expressed in this article are those of the author and do not necessarily reflect the official policy or position of the Department of the Navy, Department of Defense, nor the U.S. Government. I am a military Service member. This work was prepared as part of my official duties. Title 17, U.S.C., § 105 provides that copyright protection under this title is not available for any work of the U.S. Government. Title 17, U.S.C., § 101 defines a U.S. Government work as a work prepared by a military Service member or employee of the U.S. Government as part of that person's official duties.

## Footnotes

1 At sea level, atmospheric or barometric pressure = 1 ATA = 760 mm Hg = 0.101 MPa, and PO_2_ = 0.21 ATA, 159.6 mm Hg and 0.021 MPa.

2 Bert's book was translated from French to English and published in 1943 to aid Allied physiologists tasked with researching medical problems that limited aviation and undersea military operations during World War II [117]. The book was translated by Mary Alice (Rines) Hitchcock, a former professor of romance languages at Buchtel College (Akron, OH) and her husband, Dr. Fred A. Hitchcock (1889–1980), a member of the Department of Physiology at Ohio State University (Columbus, OH). Fred Hitchcock was the director of the OSU Laboratory of Aviation Physiology where he pioneered wartime investigations on the physiology of explosive decompression during high-altitude pressurized flight. A digital copy of the Hitchcocks' translation of Bert's tome can be downloaded at https://archive.org/details/barometricpressu00bert (downloaded 1/18/2019). The Hitchcocks' translation was republished in 1978 by the Undersea & Hyperbaric Medical Society. Today, the Aerospace Physiological Society awards three major career achievement awards annually, two of which include the Paul Bert Award (est. 1969) for outstanding research contributions in aerospace physiology, and the Fred A. Hitchcock Award (est. 1972) for career contributions by a senior aerospace physiologist for excellence in either operational aerospace physiology or aerospace physiology research; http://www.aerospacephysiologysociety.org/socirty-awards/ (downloaded 1/18/2019).

3 https://www.youtube.com/watch?reload=9&v=z7Hi0HO24Vk; YouTube video of a U.S. Navy CNS oxygen toxicity hit during World War II, presumably from the study at the USN Experimental Diving Unit in Washington, D.C., by Yarbrough et al. [191]. The clock in the lower left corner indicates that seizures begin approximately 20 min after initiating O_2_ breathing. The P_B_ and P_I_O_2_ were not indicated (downloaded 1/18/2019).

4 Neurological seizures occur in both anesthetized and unanesthetized animals. The latency to seizure becomes shorter with increasing F_I_O_2_ and/or increasing P_B_ (i.e., increasing oxygen concentration product = P_I_O_2_ × time). In rats, seizures occur after breathing 3 ATA O_2_ for 3–5 h, 5 ATA O_2_ for 5–90 min, and 6 ATA O_2_ for <20 min [68]. Typically, seizures are induced in unanesthetized animals using 4 to 5 ATA O_2_ to avert confounding symptoms of pulmonary oxygen toxicity, whereas seizures are induced in anesthetized animals using higher levels of P_I_O_2_ (6 ATA) to offset the effects of anesthesia [80].

## References

[1] Acott C. (1999). Oxygen toxicity. A brief history of oxygen in diving. SPUMS J..

[2] Ari C., Koutnik A.P., DeBlasi J., Landon C.S., Rogers C.Q., Vallas J. (2019). Delaying latency to hyperbaric oxygen-induced CNS oxygen toxicity seizures by combinations of exogenous ketone supplements. Physiol. Rep..

[3] Arieli R. (1998). Latency of oxygen toxicity of the central nervous system in rats as a function of carbon dioxide production and partial pressure of oxygen. Eur. J. Appl. Physiol. Occup. Physiol..

[4] Arieli R., Ertracht O., Oster I., Vitenstein A., Adir Y. (2005). Effects of nitrogen and helium on CNS oxygen toxicity in the rat. J. Appl. Physiol..

[5] Arieli R., Truman M., Abramovich A. (2008). Recovery from central nervous system oxygen toxicity in the rat at oxygen pressures between 100 and 300 kPa. Eur. J. Appl. Physiol..

[6] Arieli Y., Eynan M., Gancz H., Arieli R., Kashi Y. (2003). Heat acclimation prolongs the time to central nervous system oxygen toxicity in the rat: possible involvement of HSP72. Brain Res..

[7] Arieli Y., Kotler D., Eynan M., Hochman A. (2014). Hyperbaric oxygen preconditioning protects rats against CNS oxygen toxicity. Respir. Physiol. Neurobiol..

[8] Atochin D., Demchenko I., Astern J., Boso A., Piantadosi C., Huang P. (2003). Contributions of endothelial and neuronal nitric oxide synthases to cerebrovascular responses to hyperoxia. J. Cereb. Blood Flow Metab..

[9] Balentine J.D. (1982). Pathology of Oxygen Toxicity.

[10] Barone F.C., White R.F., Spera P.A., Ellison J., Currie R.W., Wang X. (1998). Ischemic preconditioning and brain tolerance: temporal histological and functional outcomes, protein synthesis requirement, and interleukin-1 receptor antagonist and early gene expression. Stroke.

[11] Batini C., Parma M., Ricci G., Zanchetti A. (1954). Mecanismi piramidali ed extrapiramidali delle convulsioni iperossiche. Arch. Fisiol..

[12] Bean J.W. (1945). Effects of oxygen at increased pressure. Physiol. Rev..

[13] Bean J.W., Rottschafer G. (1938). Reflexogenic and central structures in oxygen poisoning. J. Physiol..

[14] Bean J.W., Siegfried E.C. (1945). Transient and permanent after effects of exposure to oxygen at high pressure. Am. J. Physiol..

[15] Becker H.F., Polo O., McNamara S.G., Berthon-Jones M., Sullivan C.E. (1996). Effect of different levels of hyperoxia on breathing in healthy subjects. J. Appl. Physiol..

[16] Behnke A.R., Forbes H.S., Motley E.P. (1935). Circulatory and visual effects of oxygen at 3 atmospheres pressure. Am. J. Physiol..

[17] Behnke A.R., Johnson F.S., Poppen J.R., Motley E.P. (1935). The effect of oxygen on man at pressures from 1 to 4 atmospheres. Am. J. Physiol..

[18] Bert P. (1878). La pression barometrique. Recherches de physiologie experimentelle.

[19] Bertharion G., Barthelemy L. (1964). Effet aigu de l'oxygene hyperbare. Etude neurophysiologique. Agressologie.

[20] Bitterman N. (2004). CNS oxygen toxicity. Undersea Hyperbaric Med..

[21] Bitterman N., Bitterman H. (1998). L-arginine-NO pathway and CNS oxygen toxicity. J. Appl. Physiol..

[22] Bitterman N., Laor A., Melamed Y. (1987). CNS oxygen toxicity in oxygen-inert gas mixtures. Undersea Biomed. Res..

[23] Bitterman N., Melamed Y., Ben-Amotz A. (1994). Beta-carotene and CNS oxygen toxicity in rats. J. Appl. Physiol..

[24] Bitterman N., Samuni A. (1995). Nitroxide stable radicals protect against hyperoxic induced seizures in rats. Undersea Hyperbaric Med..

[25] Bitterman N., Samuni A. (1998). Albumin conjugated nitroxide protects against CNS oxygen toxicity in rats. Paper Presented at the Proceedings of the Annual Meeting of the EUBS on Diving and Hyperbaric Medicine.

[26] Bitterman N., Schaal S. (1995). Caffeine attenuates CNS oxygen toxicity in rats. Brain Res..

[27] Bitterman N., Skapa E., Gutterman A. (1997). Starvation and dehydration attenuate CNS oxygen toxicity in rats. Brain Res..

[28] Blackburn J.G., Ogilvie R.W., Balentine D.J. (1977). Effects of hyperbaric oxygenation on electrical activity of globus pallidus and neostriatum. Exp. Neurol..

[29] Blankenship R.E., Hartman H. (1998). The origin and evolution of oxygenic photosynthesis. TIBS.

[30] Borison H.L., Borison R., McCarthy L.E. (1984). Role of the area postrema in vomiting and related functions. Fed. Proc..

[31] Boulant J.A., Dean J.B. (1986). Temperature receptors in the central nervous system. Annu. Rev. Physiol..

[32] Bozanic J.E. (2002). Physiology Mastering Rebreathers.

[33] Bragin A., Wilson C.L., Almajano J., Mody I., Engel J. (2004). High-frequency oscillations after status epilepticus: epileptogenesis and seizure genesis. Epilepsia.

[34] Brauer R.W., Beaver R.W. (1982). Synergism of hyperoxia and high helium pressures in the causation of convulsions. J. Appl. Physiol..

[35] Bredt D.S. (1999). Endogenous nitric oxide synthesis: biological functions and pathophysiology. Free Radical Res..

[36] Browning R., Maggio R., Sahibzada N., Gale K. (1993). Role of brainstem structures in seizures initiated from the deep prepiriform cortex of rats. Epilepsia.

[37] Browning R.A. (1985). Role of the brain-stem reticular formation in tonic-clonic seizures: lesion and pharmacological studies. Fed. Proc..

[38] Butler F.K., Thalmann E.D. (1986). Central nervous system oxygen toxicity in closed circuit scuba divers II. Undersea Biomed. Res..

[39] Butler F. (2004). Closed-circuit oxygen diving in the U.S. navy. Undersea Hyperbaric Med..

[40] Butler F.K., Knafelc M.E. (1986). Screening for oxygen intolerance in U.S. navy divers. Undersea Biomed. Res..

[41] Butler F.K., Thalmann E.D. (1986). Central nervous system oxygen toxicity in closed circuit scuba divers II. Undersea Biomed. Res..

[42] Camporesi E. (1996). Hyperbaric Oxygen Therapy: a Committee Report.

[43] Carpenter M.B., Sutin J. (1983). The Medulla/The Pons Human Neuroanatomy.

[44] Castle M., Comoli E., Loewy A. (2005). Autonomic brainstem nuclei are linked to the hippocampus. Neuroscience.

[45] Cataldi M., Avoli M., de Villers-Sidani E. (2013). Resting state networks in temporal lobe epilepsy. Epilepsia.

[46] Chavko M., Auker C.R., McCarron R.M. (2003). Relationship between protein nitration and oxidation and development of hyperoxic seizures. Nitric Oxide.

[47] Chavko M., Braisted J.C., Harabin A.L. (1999). Attenuation of brain hyperbaric oxygen toxicity by fasting in not related to ketosis. Undersea Hyperbaric Med..

[48] Chavko M., McCarron R.M. (2006). Extension of brain tolerance to hyperbaric O_2_ by intermittent air breaks is related to the time of CBF increase. Brain Res..

[49] Chavko M., Xing G.Q., Keyser D.O. (2001). Increased sensitivity to seizures in repeated exposures to hyperbaric oxygen: role of NOS activation. Brain Res..

[50] Ciarlone G.E., Dean J.B. (2016). Normobaric hyperoxia stimulates superoxide and nitric oxide production in the caudal solitary complex or rat brain slices. Am. J. Cell Physiol..

[51] Ciarlone G.E., Dean J.B. (2016). Acute hypercapnic hyperoxia stimulates reactive species production in the caudal solitary complex of rat brain slices but does not induce oxidative stress. Am. J. Cell Physiol..

[52] Ciarlone G.E., Dean J.B. (2016). Hyperoxia increases neuronal responsiveness to hypercapnic acidosis (HA) in caudal solitary complex neurons in rat medullary tissue slices. J. Physiol..

[53] Ciorba M.A., Heinemann S.H., Weissbach H., Brot N., Hoshi T. (1997). Modulation of potassium channel function by methionine oxidation and reduction. Proc. Natl. Acad. Sci. U. S. A..

[54] Ciorba M.A., Heinemann S.H., Weissbach H., Brot N., Hoshi T. (1999). Regulation of voltage‐dependent K^+^ channels by methionine oxidation: effect of nitric oxide and vitamin C. FEBS (Fed. Eur. Biochem. Soc.) Lett..

[55] Ciriello J., Hochstenbach S.L., Roder S. (1994). Central Projections of Baroreceptor and Chemoreceptor Afferent Fibers in the Rat Nucleus of the Solitary Tract.

[56] Ciriello J., McMurray J.C., Babic T., de Oliveira C.V. (2003). Collateral axonal projections from hypothalamic hypocretin neurons to cardiovascular sites in nucleus ambiguus and nucleus tractus solitarius. Brain Res..

[57] Clark J.M., Lambertsen C.J., Gelfand R., Troxel A.B. (2006). Optimization of oxygen tolerance extension in rats by intermittent exposure. J. Appl. Physiol..

[58] Clarke D., Neuman T.S., Thom S.R. (2008). History of hyperbaric therapy. Physiology and Medicine of Hyperbaric Oxygen Therapy.

[59] Cohn R., Gersh I. (1945). Changes in brain potentials during convulsions induced by oxygen under pressure. J. Neurophysiol..

[60] Colton C.A., Colton J.S. (1985). Blockade of hyperbaric oxygen induced seizures by excitatory amino acid antagonists. Candian J. Physiol. Pharmacol..

[61] Cui Z.J., Han Z.Q., Li Z.Y. (2012). Modulating protein activity and cellular function by methionine residue oxidation. Amino Acids.

[62] D'Agostino D.P., Colomb D.G., Dean J.B. (2009). Effects of hyperbaric gases on membrane nanostructure and function in neurons. J. Appl. Physiol..

[63] D'Agostino D.P., Pilla R., Held H.E., Landon C.S., Puchowicz M., Brunengraber H. (2013). Therapeutic ketosis with ketone ester delays central nervous system oxygen toxicity seizures in rats. Am. J. Physiol. Regul. Integr. Comp. Physiol..

[64] D'Agostino D.P., Putnam R.W., Dean J.B. (2007). Superoxide (O_2_^-^) production in CA1 neurons of rat hippocampal slices exposed to graded levels of oxygen. J. Neurophysiol..

[65] Davis R.H. (1995). Chapter 1, A summary of the present state of the art of deep diving *Deep Diving and Submarine Operations*. A Manual for Deep Sea Divers and Compressed Air Workers.

[66] Dean J.B. (2010). Hypercapnia causes cellular oxidation and nitrosation in addition to acidosis: implications for CO_2_ chemoreceptor function and dysfunction. J. Appl. Physiol..

[67] Dean J.B. (2011). Theory of gastric CO_2_ ventilation and its control during respiratory acidosis: implications for central chemosensitivity, pH regulation, and diseases causing chronic CO_2_ retention. Respir. Physiol. Neurobiol..

[68] Dean J.B., Mulkey D.K., Garcia A.J., Putnam R.W., Henderson R.A. (2003). Neuronal sensitivity to hyperoxia, hypercapnia and inert gases at hyperbaric pressures. J. Appl. Physiol..

[69] Dean J.B., Mulkey D.K., Henderson R.A., Potter S.J., Putnam R.W. (2004). Hyperoxia, reactive O_2_ species, and hyperventilation: O_2_-sensitivity of brain stem neurons. J. Appl. Physiol..

[70] Dean J.B., Putnam R.W. (2010). The caudal solitary complex is a site of central CO_2_ chemoreception and integration of multiple systems that regulate expired CO_2_. Respir. Physiol. Neurobiol..

[71] Demchenko I., Boso A., Whorton A., Piantadosi C. (2001). Nitric oxide production is enhanced in rat brain before oxygen-induced convulsions. Brain Res..

[72] Demchenko I., Gutsaeva D.R., Moskvin A., Zhilyaev S.Y. (2010). Involvement of extracellular superoxide dismutase in regulating brain blood flow. Neurosci. Behav. Physiol..

[73] Demchenko I., Piantadosi C. (2006). Nitric oxide amplifies the excitatory to inhibitory neurotransmitter imbalance accelerating oxygen seizures. Undersea Hyperbaric Med..

[74] Demchenko I.T., Atochin D.N., Boso A.E., Astern J., Huang P.L., Piantadosi C.A. (2003). Oxygen seizure latency and peroxynitrite formation in mice lacking neuronal or endothelial nitric oxide synthases. Neurosci. Lett..

[75] Demchenko I.T., Boso A.E., Bennett P.B., Whorton A.R., Piantadosi C.A. (2000). Hyperbaric oxygen reduces cerebral blood flow by inactivating nitric oxide. Nitric Oxide Biol. Chem..

[76] Demchenko I.T., Boso A.E., O'Neil T.J., Bennett P.B., Piantadosi C.A. (2000). Nitric oxide and cerebral blood flow responses to hyperbaric oxygen. J. Appl. Physiol..

[77] Demchenko I.T., Boso A.E., Whorton A.R., Piantadosi C.A. (2001). Nitric oxide production is enhanced in rat brain before oxygen-induced convulsions. Brain Res..

[78] Demchenko I.T., Gasier H.G., Zhilyaev S.Y., Moskvin A.N., Krivchenko A.I., Piantadosi C.A. (2014). Baroreceptor afferents modulate brain excitation and influence susceptibility to toxic effects of hyperbaric oxygen. J. Appl. Physiol..

[79] Demchenko I.T., Luchakov Y.I., Moskvin A.N., Gutsaeva D.R., Allen B.W., Thalmann E.D. (2005). Cerebral blood flow and brain oxygenation in rats breathing oxygen under pressure. J. Cereb. Blood Flow Metab..

[80] Demchenko I.T., Moskvin A.N., Krivchenko A.I., Piantadosi C.A., Allen B.W. (2012). Nitric oxide-mediated central sympathetic excitation promotes CNS and pulmonary O_2_ toxicity. J. Appl. Physiol..

[81] Demchenko I.T., Oury T.D., Crapo J.D., Piantadosi C.A. (2002). Regulation of the brain's vascular responses to oxygen. Circ. Res..

[82] Demchenko I.T., Welty-Wolf K.E., Allen B.W., Piantadosi C.A. (2007). Similar but not the same: normobaric and hyperbaric pulmonary oxygen toxicity, the role of nitric oxide. Am. J. Physiol. Lung Cell Mol. Physiol..

[83] Demchenko I.T., Zhilyaev S.Y., Moskvin A.N., Krivchenko A.I., Piantadosi C.A., Allen B.W. (2017). Antiepileptic drugs prevent seizures in hyperbaric oxygen: a novel model of epileptiform activity. Brain Res..

[84] Demchenko I.T., Zhilyaev S.Y., Moskvin A.N., Piantadosi C.A., Allen B.W. (2011). Autonomic activation links CNS oxygen toxicity to acute cardiogenic pulmonary injury. Am. J. Physiol. Lung Cell Mol. Physiol..

[85] Dexter J.D., Hof D.G., Mengel C.E. (1972). Effect of sleep-wake reversal and sleep deprivation on the circadian rhythm of oxygen toxicity susceptibility. Aero. Med..

[86] Donald K. (1992). Oxygen and the Diver.

[87] Donald K. (1992). Oxygen Poisoning Studies 1942-5 Oxygen and the Diver.

[88] Donald K.W. (1947). Oxygen poisoning in man, part I. Br. Med. J..

[89] Donald K.W. (1947). Oxygen poisoning in man, part II. Br. Med. J..

[90] Droge W. (2001). Free radicals in the physiological control of cell function. Physiol. Rev..

[91] Elayan I.M., Axley M.J., Prasad P.V., Ahlers S.T., Auker C.R. (2000). Effect of hyperbaric oxygen treatment on nitric oxide and oxygen free radicals in rat brain. J. Neurophysiol..

[92] Evans M., Cogan K.E., Egan B. (2017). Metabolism of ketone bodies during exercise and training: physiological basis for exogenous supplementation. J. Physiol..

[93] Fenton L.H., Beck G., Djali S., Robinson M.B. (1993). Hypothermia induced by hyperbaric oxygen is not blocked by serotonin antagonists. Pharmacol. Biochem. Behav..

[94] Ferland R.J., Applegate C.D. (1998). The role of the ventromedial nucleus of the hypothalamus in epileptogenesis. Neuroreport.

[95] Freiberger J.J., Suliman H.B., Sheng H., McAdoo J., Piantadosi C.A., Warner D.S. (2006). A comparison of hyperbaric oxygen versus hypoxic cerebral preconditioning in neonatal rats. Brain Res..

[96] Garcia A.J., Putnam R.W., Dean J.B. (2010). Hyperbaric hyperoxia and normobaric reoxygenation increase excitability and activate oxygen-induced potentiation in CA1 hippocampal neurons. J. Appl. Physiol..

[97] Garcia A.J., Putnam R.W., Dean J.B. (2010). Hyperoxic stimulation of synchronous orthodromic activity and induction of neural plasticity does not require changes in excitatory synaptic transmission. J. Appl. Physiol..

[98] Gasier H.G., Demchenko I.T., Allen B.W., Piantadosi C.A. (2015). Effects of striatal nitric oxide production on regional cerebral blood flow and seizure development in rats exposed to extreme hyperoxia. J. Appl. Physiol..

[99] Gasier H.G., Demchenko I.T., Tatro L.G., Piantadosi C.A. (2017). S-nitrosylation of GAD65 is implicated in decreased GAD activity and oxygen-induced seizures. Neurosci. Lett..

[100] Gasier H.G., Demchenko I.T., Zhilyaev S.Y., Moskvin A.N., Krivchenko A.I., Piantadosi C.A. (2018). Adrenoceptor blockade modifies regional cerebral blood flow responses to hyperbaric hyperoxia: protection against CNS oxygen toxicity. J. Appl. Physiol..

[101] Gennser M., Blogg S.L. (2008). Oxygen or carbogen breathing before simulated submarine escape. J. Appl. Physiol..

[102] Gourine A.V., Funk G.D. (2017). On the existence of a central respiratory oxygen sensor. J. Appl. Physiol..

[103] Gowdey C.W., Patel Y.J., Stavraky G.W. (1965). Brain lesions and hyperbaric oxygen convulsions. Int. J. Neuropsychiatry.

[104] Gutsaeva D.R., Moskvin A.N., Zhilyaev S.Y., Kostkin V.B., Demchenko I.T. (2005). The roles of nitric oxide and carbon dioxide gas in the neurotoxic actions of oxygen under pressure. Neurosci. Behav. Physiol..

[105] Guzy R.D., Schumacker P.T. (2006). Oxygen sensing by mitochondria at complex III: the paradox of increased reactive oxygen species during hypoxia. Exp. Physiol..

[106] Hall A.A., Young C., Bodo M., Mahon R.T. (2013). Vigabatrin prevents seizure in swine subjected to hyperbaric hyperoxia. J. Appl. Physiol..

[107] Hamilton R.W. (1997). Rebreather physiology. SPUMS J..

[108] Hampson N., Atik D. (2003). Central nervous system oxygen toxicity during routine hyperbaric oxygen therapy. Undersea Hyperbaric Med..

[109] Harabin A.L., Braisted J.C., Flynn E.T. (1985). Response of antioxidant enzymes to intermittent and continuous hyperbaric oxygen. J. Appl. Physiol..

[110] Harel D., Kerem D., Lavy S. (1969). The influence of high oxygen pressure on the electrical activity of the brain. Electroencephalogr. Clin. Neurophysiol..

[111] Held H.E., Pilla R., Ciarlone G.E., Landon C.S., Dean J.B. (2014). Female rats are more susceptible to central nervous system oxygen toxicity than male rats. Physiol. Rep..

[112] Hesser C.M., Fagraeus L., Adolfson J. (1978). Roles of nitrogen, oxygen, and carbon dioxide in compressed-air narcosis. Undersea Biomed. Res..

[113] Hild M., Schmidt R. (1999). The mechanism of the collision-induced enhancement of the a1Δg→ X3Σg-and b1Σg+→ a1ΔgRadiative transitions of O_2_. J. Phys. Chem. A.

[114] Hilton J., Brown G., Proctor P. (1980). Effects of superoxide dismutase and catalase on central nervous system toxicity of hyperbaric oxygen. Toxicol. Appl. Pharmacol..

[115] Hinojo C.M., Ciarlone G.E., D'Agostino D.P., Dean J.B. (2018). Ketone salts inhibit production of superoxide anions during normobaric and hyperbaric hyperoxia in rat solitary complex neurons. FASEB J..

[116] Hinojo C.M., Stavitzski N.M., Landon C.S., Dean J.B. (2017). Reevaluation of CNS oxygen toxicity seizures in male Sprague-Dawley Rats. FASEB J..

[117] Hitchcock M.A., Hitchcock F.A. (1943). Barometric Pressure. Researches in Experimental Physiology.

[118] Horn E.M., Waldrop T.G. (1998). Suprapontine control of respiration. Respir. Physiol..

[119] Jahromi S.R., Togha M., Fesharaki S.H., Najafi M., Moghadam N.B., Kheradmand J.A. (2011). Gastrointestinal adverse effects of antiepileptic drugs in intractable epileptic patients. Seizure.

[120] Jamieson D., Van den Brenk A. (1963). Measurement of oxygen tensions in cerebral tissues of rats exposed to high pressures of oxygen. J. Appl. Physiol..

[121] Kadiyala S.B., Ferland R.J. (2017). Dissociation of spontaneous seizures and brainstem seizure thresholds in mice exposed to eight flurothyl-induced generalized seizures. Epilepsia Open.

[122] Kerem D.H., Geva A.B. (2005). Forecasting epilepsy from the heart rate signal. Med. Biol. Eng. Comput..

[123] Kim G., Weiss S.J., Levine R.L. (2014). Methionine oxidation and reduction in proteins. Biochim. Biophys. Acta (BBA) Gen. Subj..

[124] Kim H.-Y. (2013). The methionine sulfoxide reduction system: selenium utilization and methionine sulfoxide reductase enzymes and their functions. Antioxidants Redox Signal..

[125] King G.L., Parmemtier J.L. (1983). Oxygen toxicity of hippocampal tissue in vitro. Brain Res..

[126] Lambertsen C.J. (1988). Extension of oxygen tolerance in man: philosophy and significance. Exp. Lung Res..

[127] Lambertsen C.J. (2002). O_2_ under H_2_O. Hist. Diver.

[128] Larsen F.J., Schiffer T.A., Weitzberg E., Lundberg J.O. (2012). Regulation of mitochondrial function and energetics by reactive oxygen species. Free Radical Biol. Med..

[129] Lawrence C.H. (1996). A diving fatality due to oxygen toxicity during a “technical” dive. Med. J. Aust..

[130] Lipton J.M. (1973). Thermosensitivity of medulla oblongata in control of body temperature. Am. J. Physiol..

[131] Maalouf M., Rho J.M., Mattson M.P. (2009). The neuroprotective properties of calorie restriction, the ketogenic diet, and ketone bodies. Brain Res. Rev..

[132] Macdonald A.G., Fraser P.J. (1999). The transduction of very small hydrostatic pressures. Comp. Biochem. Physiol., A.

[133] Mahon R.T., Dainer H.M., Gibellato M.G., Soutiere S.E. (2009). Short oxygen prebreathe periods reduce or prevent severe decompression sickness in a 70-kg swine saturation model. J. Appl. Physiol..

[134] Martins I.L., Nunes J., Charneira C., Morello J., Pereira S.A., Telo J.P. (2018). The first-line antiepileptic drug carbamazepine: reaction with biologically relevant free radicals. Free Radical Biol. Med..

[135] Matott M.P., Ciarlone G.E., Putnam R.W., Dean J.B. (2014). Normobaric hyperoxia (95% O_2_) stimulates CO_2_-sensitive and CO_2_-insensitive neurons in the caudal solitary complex of rat medullary tissue slices maintained in 40% O_2_. Neuroscience.

[136] Minaev B.F., Kobzev G.I. (2003). Response calculations of electronic and vibrational transitions in molecular oxygen induced by interaction with noble gases. Spectrochim. Acta, Part A.

[137] Mula M., Trimble M.R. (2009). Antiepileptic drug-induced cognitive adverse effects. CNS Drugs.

[139] Mulkey D.K., Henderson R.A., Olson J.E., Putnam R.W., Dean J.B. (2001). Oxygen measurements in brain stem slices exposed to normobaric hyperoxia and hyperbaric oxygen. J. Appl. Physiol..

[140] Mulkey D.K., Henderson R.A., Putnam R.W., Dean J.B. (2003). Hyperbaric oxygen and chemical oxidants stimulate CO_2_/H^+^-sensitive neurons in rat brain stem slices. J. Appl. Physiol..

[141] Mulkey D.K., Henderson R.A., Putnam R.W., Dean J.B. (2003). Pressure (≤4 ATA) increases membrane conductance and firing rate in the rat solitary complex. J. Appl. Physiol..

[142] Mulkey D.K., Henderson R.A., Ritucci N.A., Putnam R.W., Dean J.B. (2004). Oxidative stress decreases intracellular pH and Na^+^/H^+^ exchange and increases excitability of solitary complex neurons from rat brain slices. Am. J. Physiol. Cell Physiol..

[144] Navy U.S. (2016). U.S. Navy Diving Manual. (SS521-AG-PRO-010). U.S. Government Printing Office.

[145] Neuman T.S., Thom S.R. (2008). Physiology and Medicine of Hyperbaric Oxygen Therapy.

[146] Nie H., Xiong L., Lao N., Chen S., Xu N., Zhu Z. (2006). Hyperbaric oxygen preconditioning induces tolerance against spinal cord ischemia by upregulation of antioxidant enzymes in rabbits. J. Cereb. Blood Flow Metab..

[147] Oury T.D., Ho Y.S., Piantadosi C.A., Crapo J.D. (1992). Extracellular superoxide dismutase, nitric oxide, and central nervous system O_2_ toxicity. Proc. Natl. Acad. Sci. Unit. States Am..

[148] Patel M. (2004). Mitochondrial dysfunction and oxidative stress: cause and consequence of epileptic seizures. Free Radical Biol. Med..

[149] Pearson J.N., Rowley S., Liang L.-P., White A.M., Day B.J., Patel M. (2015). Reactive oxygen species mediate cognitive deficits in experimental temporal lobe epilepsy. Neurobiol. Dis..

[150] Pendergast D.R., Moon R.E., Krasney J.J., Held H.E., Zamparo P. (2015). Human physiology in an aquatic environment. Compr. Physiol..

[151] Piantadosi C.A., Tatro L.G. (1990). Regional H_2_O_2_ concentration in rat brain after hyperoxic convulsions. J. Appl. Physiol..

[152] Pilla R., Landon C.S., Dean J.B. (2013). A potential early physiological marker for CNS oxygen toxicity: hyperoxic hyperpnea precedes seizure in unanesthetized rats breathing hyperbaric oxygen. J. Appl. Physiol..

[153] Plafki C., Peters P., Almeling M., Welslau W., Busch R. (2000). Complications and side effects of hyperbaric oxygen therapy. Aviat. Space Environ. Med..

[154] Prabhakar N.R., Semenza G.L. (2015). Oxygen sensing and homeostasis. Physiology.

[155] Puglia C.D., Glauser E.M., Glauser S.C. (1974). Core temperature response of rats during exposure to oxygen at high pressure. J. Appl. Physiol..

[156] Ravati A., Ahlemeyer B., Becker A., Klumpp S., Krieglstein J. (2001). Preconditioning-induced neuroprotection is mediated by reactive oxygen species and activation of the transcription factor nuclear factor-kappaB. J. Neurochem..

[157] Ravati A., Ahlemeyer B., Becker A., Krieglstein J. (2000). Preconditioning-induced neuroprotection is mediated by reactive oxygen species. Brain Res..

[158] Reyes B.A., Posada-Quintero H.F., Bales J.R., Clement A.L., Pins G.D., Swiston A. (2014). Novel electrodes for underwater ECG monitoring. IEEE Trans. Biomed. Eng..

[159] Roberts T.R., Ciarlone G.E., Dean J.B. (2017). Singlet oxygen production in the caudodorsal medulla of rat brain slices increases during hyperoxia at normobaric pressure and following decompression from iso-oxic hyperbaric nitrogen. FASEB J..

[160] Rucci F.S., Giretti M.L., La Rocca M. (1966). Changes in electrical activity of the cerebral cortex and some subcortical centers in hyperbaric oxygen. Electroencephalogr. Clin. Neurophysiol..

[161] Sanders R.W., Katz K.D., Suyama J., Akhtar J., O'Toole K.S., Corll D. (2012). Seizure during hyperbaric oxygen therapy for carbon monoxide toxicity: a case series and five-year experience. J. Emerg. Med..

[162] Shanlin F., Stocker R., Davies M.J. (1997). Biochemistry and pathology of radical-mediated protein oxidation. Biochem. J..

[163] Sheffield P.J., Desautels D.A. (1997). Hyperbaric and hypobaric chamber fires: a 73-year analysis. Undersea Hyperbaric Med..

[164] Shih P.M. (2015). Photosynthesis and early Earth. Curr. Biol..

[165] Shmalberg J., Davies W., Lopez S., Shmalberg D., Zilberschtein J. (2015). Rectal temperature changes and oxygen toxicity in dogs treated in a monoplace chamber. Undersea Hyperbaric Med..

[166] Shykoff B. (2005). Pulmonary effects of submerged oxygen breathing: 4-, 6-, and 8-hour dives at 140 kPa. Undersea Hyperbaric Med..

[167] Smerz R.W. (2004). Incidence of oxygen toxicity during the treatment of dysbarism. Undersea Hyperbaric Med..

[168] Smith J.L. (1899). The pathological effects due to increase of oxygen tension in the air breathed. J. Physiol..

[169] Sonnenschein R.R., Stein S.N. (1953). Electrical activity of the brain in acute oxygen poisoning. Electroencephalogr. Clin. Neurophysiol..

[170] Strohm C., Barancik M., von Bruehl M., Strniskova M., Ullmann C., Zimmermann R. (2002). Transcription inhibitor actinomycin-D abolishes the cardioprotective effect of ischemic reconditioning. Cardiovasc. Res..

[171] Tanaka M., Nagashima K., McAllen R.M., Kanosue K. (2004). Role of the medullary raphe in thermoregulatory vasomotor control in rats. J. Physiol..

[172] Tang X.D., Daggett H., Hanner M., Garcia M.L., McManus O.B., Brot N. (2001). Oxidative regulation of large conductance calcium-activated potassium channels. J. Gen. Physiol..

[173] Taylor H.J. (1949). The role of carbon dioxide in oxygen poisoning. J. Physiol..

[174] Thom S.R., Bhopale V.M., Yang M. (2014). Neutrophils generate microparticles during exposure to inert gases due to cytoskeletal oxidative stress. J. Biol. Chem..

[175] Thom S.R., Marquis R.E. (1984). Microbial growth modification by compressed gases and hydrostatic pressure. Appl. Environ. Microbiol..

[176] Thom S.R., Marquis R.E. (1987). Free radical reactions and the inhibitory and lethal actions of high-pressure gases. Undersea Biomed. Res..

[177] Torbati D., Church D.F., Keller J.M., Pryor W.A. (1992). Free radical generation in the brain precedes hyperbaric oxygen-induced convulsions. Free Radical Biol. Med..

[178] Turrens J.F. (1997). Superoxide production by the mitochondrial respiratory chain. Biosci. Rep..

[179] Turrens J.F. (2003). Mitochondrial formation of reactive oxygen species. J. Physiol..

[180] Tzuk-Shina T., Bitterman N., Harel D. (1991). The effect of vigabatrin on central nervous system oxygen toxicity in rats. Eur. J. Pharmacol..

[181] UHMS (2018). Indications for hyperbaric oxygen therapy. Undersea and Hyperbaric Medical Society Hyperbaric Oxygen Therapy Indications*.* 13^th^.

[182] USN (2014). SSN 688 Class Guard Book, Disabled Submarine Survival Guide, Aft Compartment. (S9594-AP-SAR-A10 REV 3).

[183] Vann R.D. (1988). Oxygen Toxicity Risk Assessment.

[184] Vann R.D. (2004). Lambertsen and O_2_: beginnings of operational physiology. Undersea Hyperbaric Med..

[185] Veech R.L. (2004). The therapeutic implications of ketone bodies: the effects of ketone bodies in pathological conditions: ketosis, ketogenic diet, redox states, insulin resistance, and mitochondrial metabolism. Prostaglandins Leukot. Essent. Fatty Acids.

[186] Verrotti A., Laus M., Coppola G., Parisi P., Mohn A., Chiarelli F. (2010). Catamenial epilepsy: hormonal aspects. Gynecol. Endocrinol..

[187] Viggiano A., Pilla R., Arnold P., Monda M., D'Agostino D., Coppola G. (2015). Anticonvulsant properties of an oral ketone ester in a pentylenetetrazole-model of seizure. Brain Res..

[188] Viggiano A., Stoddard M., Pisano S., Operto F.F., Iovane V., Monda M. (2016). Ketogenic diet prevents neuronal firing increase within the substantia nigra during pentylenetetrazole-induced seizure in rats. Brain Res. Bull..

[189] Vismer M.S., Forcelli P.A., Skopin M.D., Gale K., Koubeissi M.Z. (2015). The piriform, perirhinal, and entorhinal cortex in seizure generation. Front. Neural Circuits.

[190] Wardman P. (2007). Fluorescent and luminescent probes for measurement of oxidative and nitrosative species in cells and tissues: progress, pitfalls, and prospects. Free Radical Biol. Med..

[191] Yarbrough O.D., Welham W., Brinton E.S., Behnke A.R. (1947). Symptoms of Oxygen Poisoning and Limits of Tolerance at Rest and at Work Washington, D.C.: Naval Experimental Diving Unit (NEDU)-47-01. United States Navy Experimental Diving Unit Technical Report.

[192] Yildiz S., Aktas S., Cimsit M., Ay H., Torol E. (2004). Seizure incidence in 80,000 patient treatments with hyperbaric oxygen. Aviat. Space Environ. Med..

[193] Yusa T., Beckman J.S., Crapo J.D., Freeman B.A. (1987). Hyperoxia increases H_2_O_2_ production by brain in vivo. J. Appl. Physiol..

[194] Zhang J., Su Y., Oury T.D., Piantadosi C.A. (1993). Cerebral amino acid, norepinephrine and nitric oxide metabolism in CNS oxygen toxicity. Brain Res..

